# A Sporulation-Independent Way of Life for Bacillus thuringiensis in the Late Stages of an Infection

**DOI:** 10.1128/mbio.00371-23

**Published:** 2023-04-27

**Authors:** Hasna Toukabri, Didier Lereclus, Leyla Slamti

**Affiliations:** a Micalis Institute, INRAE, AgroParisTech, Université Paris-Saclay, Jouy-en-Josas, France; Swammerdam Institute for Life Sciences, University of Amsterdam; Institut Pasteur

**Keywords:** *Bacillus*, adaptation, infection, nonsporulated, oxidative stress, persistence

## Abstract

The formation of endospores has been considered the unique survival and transmission mode of sporulating *Firmicutes* due to the exceptional resistance and persistence of this bacterial form. However, nonsporulated bacteria (Spo^–^) were reported at the early stages following the death of a host infected with Bacillus thuringiensis, an entomopathogenic sporulating bacterium. Here, we investigated the characteristics of the bacterial population in the late stages of an infection in the B. thuringiensis/Galleria mellonella infection model. Using fluorescent reporters and molecular markers coupled to flow cytometry, we demonstrated that the Spo^–^ cells persist and constitute about half of the population 2 weeks post-infection (p.i.). Protein synthesis and growth recovery assays indicated that they are in a metabolically slowed-down state. These bacteria were extremely resistant to the insect cadaver environment, which did not support growth of *in vitro*-grown vegetative cells and spores. A transcriptomic analysis of this subpopulation at 7 days p.i. revealed a signature profile of this state, and the expression analysis of individual genes at the cell level showed that more bacteria mount an oxidative stress response as their survival time increases, in agreement with the increase of the free radical level in the host cadaver and in the number of reactive oxygen species (ROS)-producing bacteria. Altogether, these data show for the first time that nonsporulated bacteria are able to survive for a prolonged period of time in the context of an infection and indicate that they engage in a profound adaptation process that leads to their persistence in the host cadaver.

## INTRODUCTION

The ability of bacteria to face environmental fluctuations is essential to their survival. These stresses include nutrient limitation or depletion, pH variations, temperature, oxygen level, osmolarity, radiation, and chemical exposure. In conditions that do not support their growth, some *Firmicutes*, such as *Bacillus* species, have evolved the ultimate mode of resistance, sporulation (1). This survival mode requires the activation of a specific and complex developmental pathway that leads to the formation of a spore, a highly resistant dormant cell that allows these species to survive in extreme conditions for extended periods (2–5).

Due to their resistant nature, the formation of endospores was considered the unique mode of survival and transmission of these sporulating species. However, it was recently shown that a Bacillus subtilis sporulation mutant was able to survive deep starvation for several months by entering an oligotrophic state that supports extreme slow growth (6). Moreover, spontaneous mutants in *spo0A*, the gene specifying the master regulator of sporulation (7, 8), were isolated alongside spores after a month-long incubation of the foodborne pathogen Bacillus cereus under abiotic conditions in groundwater (9). Furthermore, a B. cereus strain impaired in sporulation was shown to persist for several days on ready-to-eat vegetables and retained its ability to cause illness in a mammalian host (10). In addition, it was shown that spores represented only 30% of the bacterial population that survived in an insect cadaver 4 days post-infection (dpi) with Bacillus thuringiensis and that a sporulation mutant was able to survive in the cadaver during that time (11).

B. thuringiensis is an entomopathogenic bacterium that belongs to the B. cereus sensu lato group comprising several species that are pathogenic to humans and animals, notably B. cereus sensu stricto, which is responsible for foodborne toxi-infections as well as systemic infections, and Bacillus anthracis, the agent of anthrax (12). B. thuringiensis was shown to carry out a full infectious cycle in the Galleria mellonella insect larva model. This process is composed of three major phases controlled by quorum-sensing systems (13): virulence that allows the bacteria to invade and kill the host (14), necrotrophism which permits the bacteria to feed on the cadaver (11), and sporulation. These processes were reported in insects using fluorescent reporters to detect the activity of the regulators responsible for these states at the cell level (15). Sporulation was shown to occur only in the part of the subpopulation that had activated the necrotrophic regulon. This is due to the dual nature of the NprR regulator, which acts as an activator of the necrotrophic regulon when bound to its cognate signaling peptide NprX and as a phosphatase negatively regulating sporulation in its apo-form (16). Necrotrophism was reported to be activated during the whole time span of the experiment (i.e., 4 days) in bacteria that did not engage in sporulation. A category of cells which did not express the necrotrophic or the sporulation reporters in the host was identified and designated Nec^–^/Spo^–^ (15, 17). Most of these bacteria were identified as viable using a cell death marker at 3 dpi, and the results suggested that they were not in the exponential or stationary phase (18 and our unpublished data).

In this study, we investigated the composition of the bacterial population during the late stages of an infection as well as the characteristics and the proportion of the nonsporulated bacterial subpopulation in the B. thuringiensis/G. mellonella infection model. We used an unstable fluorescent reporter coupled to flow cytometry to determine the actual duration of necrotrophism. We also examined the metabolic state of the Spo^–^ cells using protein synthesis and growth recovery assays on rich or insect-based medium. A transcriptomic analysis of the Spo^–^ subpopulation at 7 dpi revealed a signature profile of this state, highlighting an increased number of upregulated oxidative stress response genes. We then monitored the expression kinetics of these genes at the scale of the cell and assayed the level of free radicals in the cadaver and inside the bacteria during infection to examine the environmental stress encountered by these cells.

## RESULTS

### Nonsporulated bacteria survive in insect cadavers for at least 14 days post-infection.

**FIG 1:**
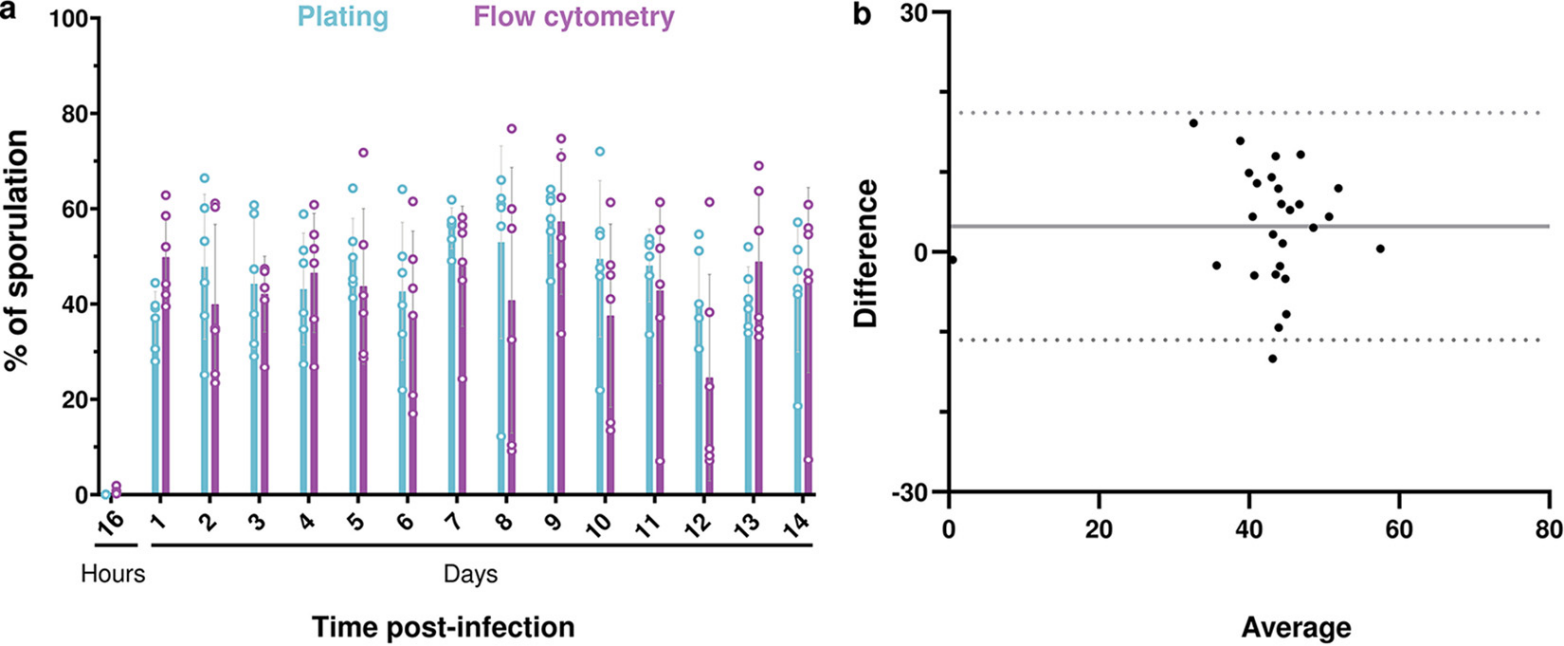
Monitoring of the sporulated subpopulation during long-term infection. (a) The percentage of sporulation was determined daily for 14 days after intrahemocoelic infection of G. mellonella with Bt (pP*nprA′gfp_Bte_AAV-*P*spoIIQ′mcherry*) by plating onto LB agar (blue) or by flow cytometry (purple). Six larvae were crushed at the time points indicated, and serial dilutions of the homogenate were plated directly onto LB agar for total population enumeration or after heating at 80°C for 12 min to account for heat-resistant spores. For the flow cytometry assay, the same samples were fixed, and red fluorescent events were discriminated in cytograms (as described in Materials and Methods) and counted as sporulating bacteria. The total population was determined by the number of total events. Each symbol represents the data relative to bacteria extracted from one larva. The data are the result of two independent experiments, and the error bars show the standard deviation from the mean. (b) Bland-Altman comparison plot between flow cytometry and plating sporulation assessment. The difference between plating and flow cytometry is plotted on the *y* axis, and the average of the values obtained by both methods is plotted on the *x* axis. The black line represents the bias value (3,198), and dotted lines indicate the 95% limits of agreement. Data were collected from 27 larvae.

**FIG 2:**
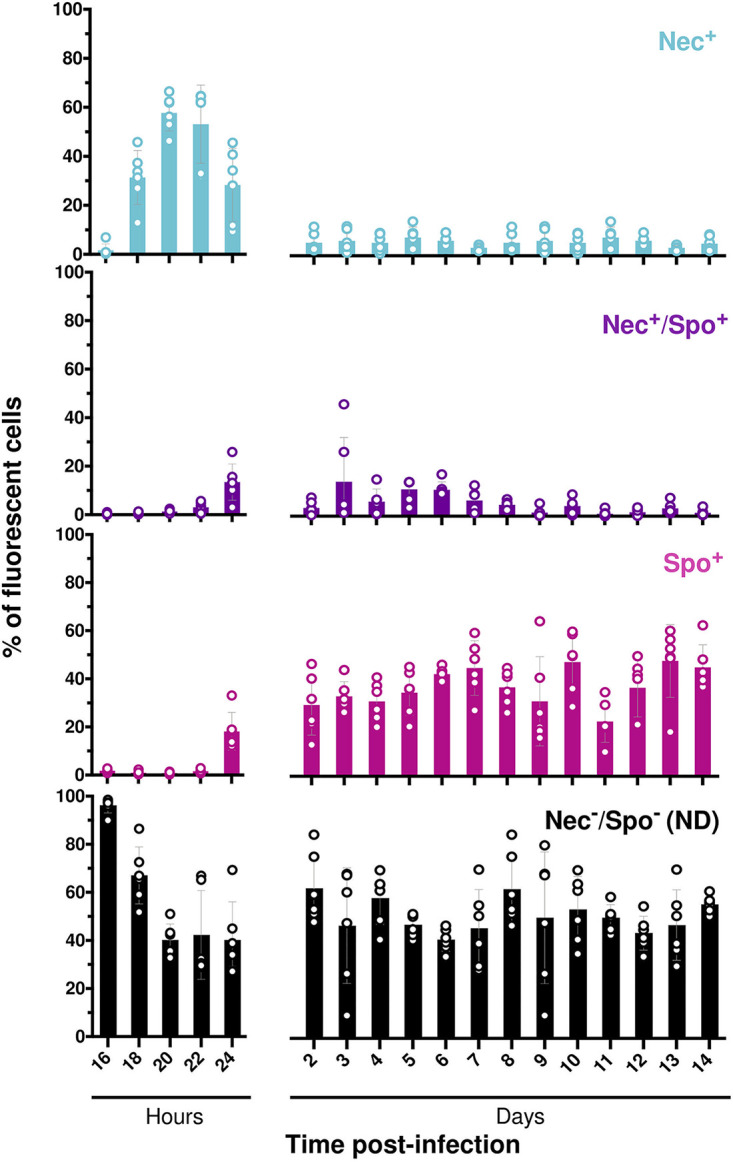
Necrotrophism and sporulation promoter activities in bacterial cells during long-term infection. Flow cytometry analysis of Bt (pP*nprA′gfp_Bte_AAV-*P*spoIIQ′mcherry*) cells during G. mellonella infection. Cells were extracted from 6 larva cadavers at the time points indicated for 14 days after intrahemocoelic infection. The percentage of the total cell count represented by each population was discriminated in cytograms and is presented as a function of time (as described in Materials and Methods). Each population phenotype is associated with a color: Nec^+^ in blue for cells expressing the necrotrophic marker only, Spo^+^ in pink for cells expressing the sporulation marker only, Nec^+^/Spo^+^ in purple for cells expressing both markers, ND in black for cells that do not express any of the markers used. Each symbol represents bacteria extracted from one larva. The data are the result of two independent experiments, and the error bars show the standard deviation from the mean.

A previous study recorded the sporulation rate of B. thuringiensis in insect larva cadavers and showed that heat-resistant spores were detected 24 h post-infection (hpi) and reached a plateau at 30% of the bacterial population from 48 hpi until the end of the experiment 4 dpi (11). This result indicated that a large part of the bacterial population was able to survive for 4 days under these conditions without resorting to sporulation. In order to determine if the nonsporulating cells were able to persist during a longer period of time, and if the sporulation rate remained constant, we monitored the fate of the bacterial population for 14 days using two complementary methods. B. thuringiensis cells harboring P*spoIIQ'mcherry*, a transcriptional fusion between the promoter of *spoIIQ*, a sporulation gene activated when the cells are committed to sporulation (19), and the B. thuringiensis-optimized *mcherry* fluorescent reporter gene (15), were injected into G. mellonella larvae. Sporulation was then assayed by plating and by flow cytometry (as described in Materials and Methods). Under our conditions, flow cytometry analysis of P*spoIIQ'mcherry* cannot discriminate between bacteria irreversibly engaged in sporulation and sporulated cells, as the mCherry protein remains fluorescent in spores. In the plating assay, we cannot exclude that some spores will not be able to germinate and form colonies on LB agar plates. These two methods were therefore implemented to give the closest assessment of the sporulated and nonsporulated subpopulations. Larvae died between 8 and 12 hpi (see Fig. S1 in the supplemental material), and bacteria were extracted from insect cadavers from 16 hpi to 14 dpi for analysis. The plating assay showed that the total number of CFU per larva remained stable for 14 dpi (Fig. S2). mCherry-positive bacteria (Fig. 1a and 2) and heat-resistant spores (Fig. 1a and Fig. S2) were detected starting at 1 dpi and represented about 40% and 50% of the total population, respectively. Accordingly, microscopy results indicated that the mCherry-positive events mainly correspond to spores from the third day post-infection onward (Fig. S3 and data not shown). The sporulation rate remained stable for 14 days and was amounted to between 30% and 55%, indicating that up to 70% of the cells could be in a nonsporulated form. We cannot exclude that germination occurred in insect cadavers, as a few spores had lost their refringence and remained fluorescent when observed by microscopy (data not shown). However, less than 5% of the bacteria presented this profile (data not shown). Comparison of the data using a Bland-Altman plot (Fig. 2b) showed that the bias value, which is the mean of the differences between the sporulation percentages obtained with each method, is 3.2% and the standard deviation (SD) of bias is 7.3%. These differences are acceptable for measurements of sporulation rates and therefore show that both methods are equivalent for evaluating this process under these conditions. Flow cytometry is thus suitable to discriminate between sporulating and nonsporulating cells. Taken together, these results show that sporulation is not the only survival pathway in insect cadavers even in late-infection stages.

10.1128/mbio.00371-23.2 FIG S1 Time-lapse photography set up to determine G. mellonella lethal time. (a) The first picture shows infected larvae incubated in 6-well plates at 30°C under a Nikon CoolPix P1 camera set on time-lapse photography mode to determine the time of death for each larva. Melanization as shown by the second picture at 24 hpi and absence of movement were required to consider a larva dead. (b) G. mellonella lethal time after infection with B. thuringiensis strain 407. A picture was taken every 10 min with the time-lapse photography, and the lethal time post-infection is reported. The black line indicates the median, and the blue lines indicate the first and last quartile; *n* > 150. Download FIG S1, DOCX file, 1.7 MB. Copyright © 2023 Toukabri et al. 2023 Toukabri et al. https://creativecommons.org/licenses/by/4.0/ This content is distributed under the terms of the Creative Commons Attribution 4.0 International license.

10.1128/mbio.00371-23.3 FIG S2 Monitoring of B. thuringiensis survival during long-term infection. The total bacterial population (blue) and spores (purple) were numerated daily for 14 days after intrahemocoelic infection of G. mellonella with Bt (pP*nprA′gfp_Bte_AAV*-pP*spoIIQ′mcherry*). Larvae were crushed at the time points indicated, and serial dilutions of the homogenate were directly plated onto LB agar for total population numeration. The bacterial suspensions were heated at 80°C for 12 min and plated onto LB agar to count heat-resistant spores. Each symbol represents bacteria extracted from one larva. Three larvae were used for each experiment and time point. The data are the results of two independent experiments, and the error bars show the standard deviation from the mean. Download FIG S2, DOCX file, 0.06 MB. Copyright © 2023 Toukabri et al. 2023 Toukabri et al. https://creativecommons.org/licenses/by/4.0/ This content is distributed under the terms of the Creative Commons Attribution 4.0 International license.

10.1128/mbio.00371-23.4 FIG S3 Microscopy observations of the necrotrophism and sporulation promoter activity in bacterial cells during long-term infection. Bacteria were analyzed by fluorescence microscopy at the time points indicated. The top panels show the merge between the phase contrast and epifluorescence images channels; the bottom panels show epifluorescence images. Cells were false colored in green for Nec^+^ cells and pink for Spo^+^ cells. The scale bars represent 10 μm. Download FIG S3, DOCX file, 0.3 MB. Copyright © 2023 Toukabri et al. 2023 Toukabri et al. https://creativecommons.org/licenses/by/4.0/ This content is distributed under the terms of the Creative Commons Attribution 4.0 International license.

### Necrotrophism is a transient state occurring at early stages of survival in insect cadavers.

To assess the proportion of bacteria in the necrotrophic state during the late stages of infection in insect larvae, we monitored this state for 14 dpi using a transcriptional fusion between the promoter of *nprA*, an NprR-regulated gene (20), and the reporter gene *gfp_Bte_AAV*, encoding an unstable green fluorescent protein (GFP) (18). This reporter gene was previously used to detect vegetative cells *in vivo* and showed its relevance as a molecular tool to assess transient gene expression during infection (18). We associated this construct with the P*spoIIQ'mcherry* fusion. This combination of reporters allowed us to determine the fraction of the population that was able to persist in the late stages of infection without entering sporulation or activating the necrotrophism pathway. G. mellonella larvae were infected by intrahemocoelic injection with the B. thuringiensis (Bt) (pP*nprA*'*gfp_Bte_AAV*-P*spoIIQ'mcherry*) strain. Figure 2 shows that expression of the P*nprA'gfp_Bte_AAV* fusion started between 16 and 18 hpi with about 30% of the bacteria in the necrotrophic state (Nec^+^) at 18 hpi. The number of Nec^+^ bacteria peaked at 20 hpi to near 60% and decreased to around 30% at 24 hpi. Expression of the P*spoIIQ'mcherry* fusion started between 22 and 24 hpi with about 20% of the bacteria expressing the sporulation reporter only (Spo^+^) and 15% of the population expressing both the necrotrophic and sporulation reporters (Nec^+^/Spo^+^). At 2 dpi the total number of Nec^+^ and Nec^+^/Spo^+^ bacteria decreased to about 10% of the population and remained at that level until the end of the experiment. These data show that necrotrophism is a transient state in which the bacteria remain during a limited period after the death of their host. Furthermore, our results reveal that the previously reported proportion of Nec^–^/Spo^–^ cells was underestimated.

### The majority of the nonsporulated late-infection stage bacteria present cellular vitality.

**FIG 3:**
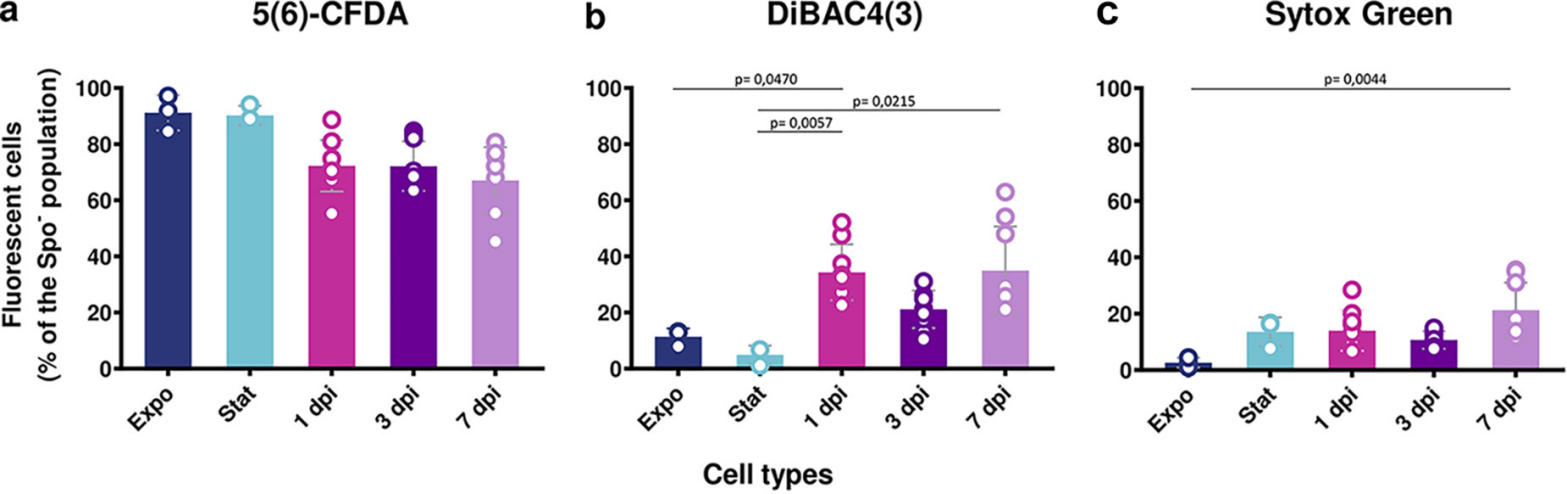
Assessment of the vitality status of the nonsporulated bacteria during infection. Flow cytometry analysis of Bt (pP*spoIIQ′mcherry*) cells grown in LB medium and harvested in exponential (OD_600_ = 1, dark blue) or stationary phase (OD_600_ = 8, light blue) or extracted from G. mellonella cadavers at 1 day (pink), 3 days (dark purple), and 7 days (light purple) post-infection. (a to c) The samples were incubated for 10 min in the dark at room temperature (RT) with 5 μM 5(6)-CFDA (a), 0,5 μM DiBAC4(3) (b), and 0.5 μM Sytox Green (c) before being subjected to analysis. The percentage of marked cells among nonsporulating bacteria discriminated in cytograms (as described in Materials and Methods) is presented as a function of time. Unmarked and heat-shocked bacteria were used as controls. Each symbol represents the data relative to bacteria extracted from one larva. The data are the result of three independent experiments, and the error bars show the standard deviation from the mean. Statistically significant differences are indicated by black bars with the *P* value obtained after a Kruskal-Wallis test followed by Dunn’s multiple-comparison test.

We analyzed the behavior and features of the Spo^–^ bacteria during infection using molecular dyes to assess bacterial vitality. We used three commercial dyes to examine different vitality features of these bacteria: enzymatic activity by assessing esterase activity with 5(6)-CFDA (Fig. 3a), membrane potential with DiBAC4(3) (Fig. 3b), and membrane integrity with Sytox Green (Fig. 3c) (21). Sytox Green staining was previously successfully used on cells extracted from insect cadavers to detect mortality among the 3 dpi nonnecrotrophic cells (18). Strain Bt (pP*spoIIQ′mcherry*) cultured in LB and harvested in the exponential-growth phase (Expo) and stationary-growth phase (Stat) or extracted from insect cadavers at 1, 3, and 7 dpi, was incubated with the molecular dyes listed above. Bacteria were then analyzed by flow cytometry (Fig. 3) to quantify the stained Spo^–^ cells. The results show that 5(6)-CFDA stained 90% of Expo and Stat cells, and 67% to 72% of bacteria extracted from insect cadavers (Fig. 3a). However, this difference was not statistically significant. Less than 16% of Expo and Stat cells and approximately 30% of bacteria extracted from insect cadavers were marked with DiBAC4(3). (Fig. 3b). The latter presented a more heterogeneous profile, as the percentage of marked cells ranged from 15% to 60% of the Spo^–^ bacteria. Finally, approximately 20% of Stat cells and bacteria extracted from insect cadavers were marked with Sytox Green, compared to less than 5% of Expo cells (Fig. 3c). Our results indicate that a portion of the Spo^–^ cells extracted from insect cadavers were damaged, but the majority of these bacteria presented esterase activity, membrane polarization, and membrane integrity, indicating cellular vitality.

### Protein production induction is delayed in nonsporulated late-infection stage bacteria.

**FIG 4:**
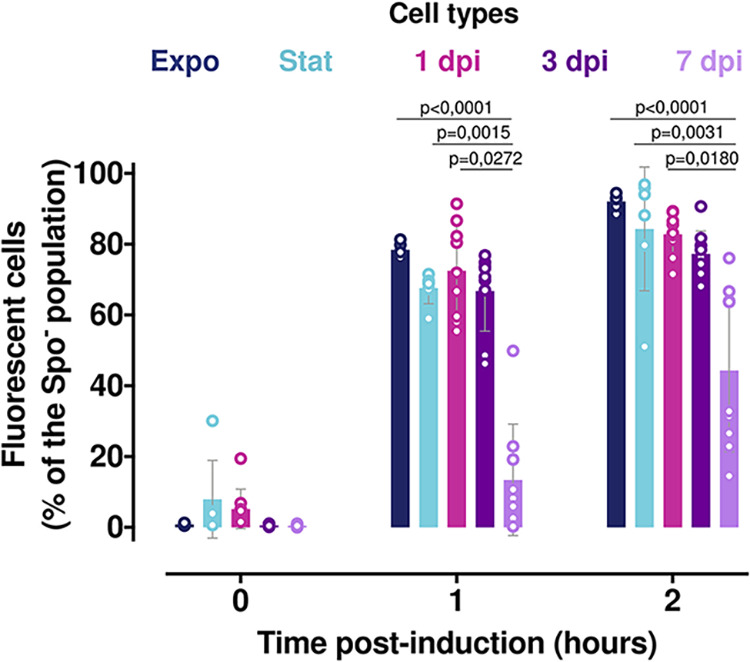
Induction of *gfp* expression in nonsporulated bacteria during infection. Flow cytometry analysis of Bt (pP*x+′gfp_Bte_-*P*spoIIQ′mcherry*) cells grown in HCT medium and harvested in exponential (OD_600_ = 1, dark blue) or stationary phase (OD_600_ = 6, light blue) or extracted from G. mellonella cadavers at 1 day (pink), 3 days (dark purple), and 7 days (light purple) post-infection. The bacteria were resuspended in conditioned HCT medium supplemented with 25 mM xylose. Aliquots were analyzed at the time of inoculation and 1 and 2 h after the start of the induction. Green fluorescent cells among the nonsporulating bacteria were discriminated in cytograms as described in Materials and Methods. Each symbol represents the data relative to bacteria extracted from one larva. The data are the result of three independent experiments, and the error bars show the standard deviation from the mean. Statistically significant differences are indicated by black bars with the *P* value obtained after a Kruskal-Wallis test followed by Dunn’s multiple-comparison test.

To further assess the physiological state of the 7-dpi bacteria, we examined the metabolic activity of these cells by recording their protein synthesis ability. We designed a strain harboring the transcriptional fusion P*x+'gfp_Bte_* between a xylose-inducible promoter and a GFP-encoding reporter gene associated with the sporulation reporter P*spoIIQ'mcherry*. We assessed the ability of the Spo^–^ cells to synthesize proteins by incubating them in conditioned HCT medium (HCTc) supplemented with xylose for 1 and 2 h and measuring the proportion of GFP-positive and mCherry-negative bacteria using flow cytometry (Fig. 4) coupled with microscopy observations (Fig. S4). Expo and Stat cells harvested from HCT cultures at 30°C, as well as bacteria extracted from insect cadavers at different time points (1, 3, and 7 dpi) were subjected to this treatment. The results show that at the start of induction, almost no bacteria expressed the P*x+'gfp_Bte_* fusion in any of the samples, except for about 6% of the Stat and 1-dpi cells (Fig. 4). However, approximately 70% of the Spo^−^ Expo, Stat, and 1- and 3-dpi cells synthesized GFP at 1 h post-induction. At 2 h post-induction the proportion of bacteria expressing *gfp_Bte_* in these samples slightly increased to 80%. Interestingly, the 7-dpi bacteria presented a different induction profile. At 1 h post-induction, less than 15% of the Spo^–^ bacteria were fluorescent, whereas at 2 h post-induction 15% to 75% of these cells were able to produce GFP. This result indicates that most of the 7-dpi bacteria retain their ability to synthesize proteins, albeit with a greater time delay and variability between samples than for the other bacterial conditions assayed. This indicates that nonsporulating bacteria undergo physiological changes during the late infection stages, suggestive of a metabolic slowdown.

10.1128/mbio.00371-23.5 FIG S4 Induction of *gfp* expression in nonsporulated bacteria. Fluorescence microscopy images at the time after induction indicated above the pictures. Bottom panels, epifluorescence images; top panels, merge between the two channels. Cells were false colored in green for GFP-expressing cells and pink for Spo^+^ cells. The scale bar represents 10 μm. These results are representative of three independent experiments. Download FIG S4, DOCX file, 0.1 MB. Copyright © 2023 Toukabri et al. 2023 Toukabri et al. https://creativecommons.org/licenses/by/4.0/ This content is distributed under the terms of the Creative Commons Attribution 4.0 International license.

### Growth recovery of late-infection stage bacteria is impaired on rich medium, in contrast to insect medium.

**FIG 5:**
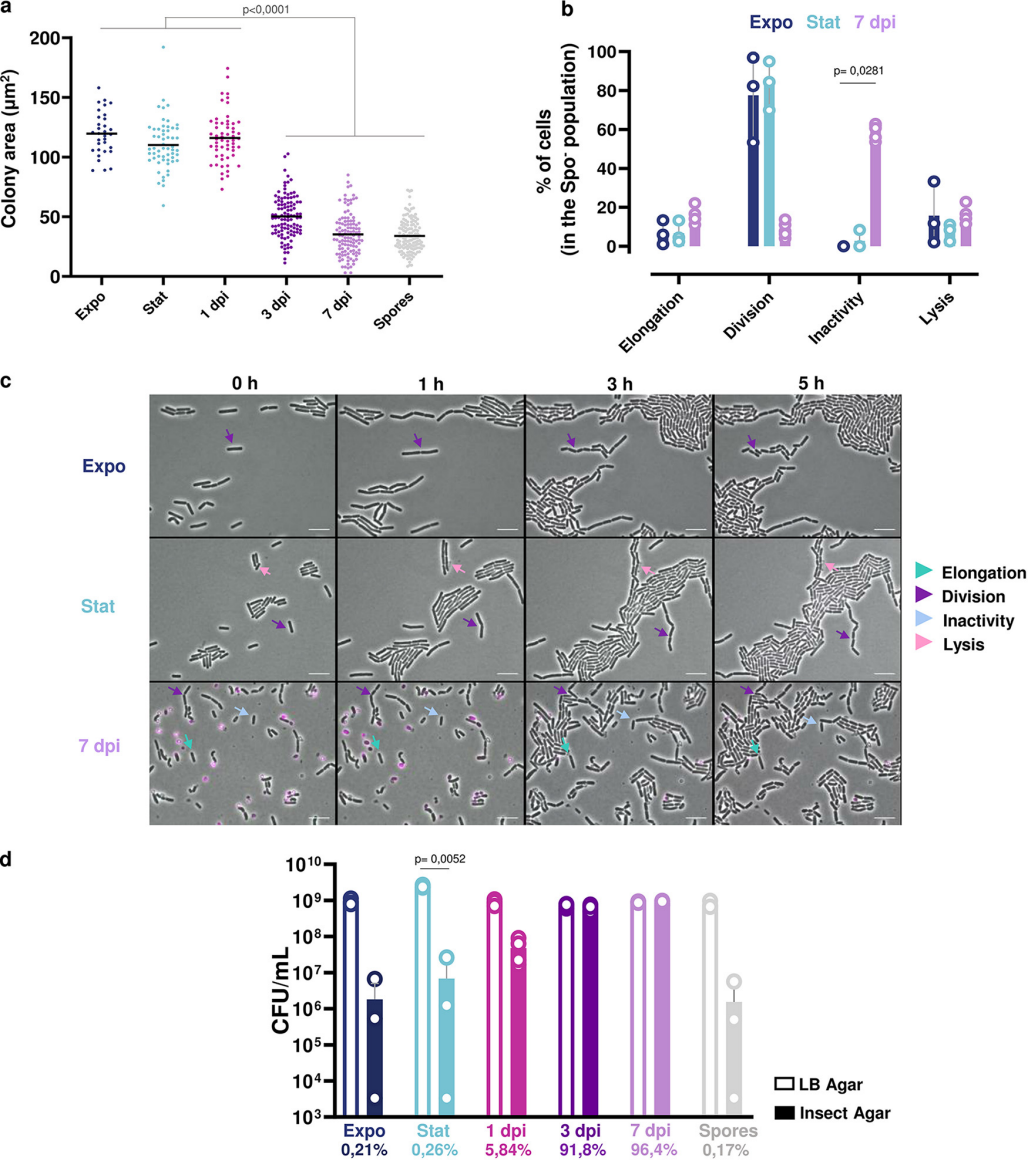
Recovery assessment of the bacteria extracted from insect cadavers. (a) Colony size analysis of Bt (pP*spoIIQ′mcherry*) cells grown in LB medium and harvested in exponential (OD_600_ = 1, dark blue) or stationary phase (OD_600_ = 8, light blue) or extracted from G. mellonella cadavers at 1 day (pink), 3 days (dark purple), and 7 days (light purple) post-infection, or of an *in vitro* spore preparation in LB medium (gray) plated on LB agar. Colonies were photographed under a binocular stereo microscope 16 h postplating. Colony size was then measured using ImageJ. At least 145 cells were measured for each condition. Each symbol on the graph represents one colony. The data are the results of three independent experiments, and the black horizontal bars indicate the mean. Statistically significant differences are indicated by black bars with the *P* value obtained after a Kruskal-Wallis test followed by Dunn’s multiple-comparison test. (b) Time-lapse microscopy analysis performed on bacteria spotted onto LB agarose pads. One picture was taken per hour for 5 h. Each nonsporulating cell was assigned to one of the following categories: Elongation, division, inactivity and lysis. The percentage of cells for each category was then calculated. The data are the result of three independent experiments. At least 145 cells were counted, and the error bars show the standard deviation from the mean. Statistically significant differences are indicated by black bars with the *P* value obtained after a Kruskal-Wallis test followed by Dunn’s multiple-comparison test. (c) Time-lapse microscopy pictures with the time scale represented above each picture. Bacteria were false colored in pink for Spo^+^ cells. Arrows point to representative bacteria of the categories listed in panel b. The scale bar represents 10 μm. These results are representative of three independent experiments. (d) Growth recovery on Insect agar medium. The bacteria were spotted onto Insect agar (filled bars) or LB agar plates (open bars). The bacteria were incubated at RT, and the CFU were enumerated the day after plating on the latter and 3 days later on the former. Each symbol represents bacteria extracted from one larva or originating from one culture. The percentage of CFU counted on LB versus Insect agar is indicated below each cell type. The data are the results of four independent experiments, and the error bars show the standard deviation from the mean.

We performed a colony-size assay to determine whether bacteria extracted at late time points from insect cadavers were able to replicate when exposed to fresh growth medium. Expo cells, Stat cells, spores harvested from 48-h LB cultures at 30°C, and bacteria extracted from insect cadavers at 1, 3, and 7 dpi were plated onto LB agar. Plates were photographed after 16 h of incubation at room temperature (Fig. S5), and colony size was determined as described in Materials and Methods. The results show that colonies resulting from 3- and 7-dpi bacteria, as well as *in vitro*-prepared spores, were smaller than those resulting from Expo, Stat, and 1-dpi cells (Fig. 5a). This indicates that late-infection stage bacteria recover more slowly than bacteria sampled at an earlier infection stage or than *in vitro*-grown vegetative cells, whereas their recovery is similar to that of *in vitro*-generated spores. The results might reflect a delay of growth due to the germination of the spores present at 3 dpi or after. However, all the colonies were smaller and therefore included the Spo^–^ cells, unless they were unable to form a colony. After longer incubation times, all the samples displayed similar colony sizes (data not shown). In order to determine more precisely the behavior of the bacterial subpopulations, we also examined the growth of 7-dpi bacteria at the single-cell level using time-lapse microscopy. Expo, Stat, and 7-dpi bacteria were inoculated on LB agarose strips at 30°C, and the behavior of Spo^–^ cells was followed for 5 h (Fig. 5b and c). About 8% to 16% of the counted bacteria lysed regardless of the sample. Approximately 80% of Expo and Stat cells were able to divide, whereas the majority of 7-dpi cells were inactive (Fig. 5b and c). We observed elongation and growth for less than 20% of the 7-dpi bacteria (Fig. 5b and c). These results show that 7-dpi bacteria rarely divided over a 5-h time-lapse experiment, which is in agreement with the slower growth recovery phenotype observed on LB plates.

10.1128/mbio.00371-23.6 FIG S5 Recovery assessment on LB agar plates. Plating of Bt (pP*spoIIQ′mcherry*) cells grown in LB medium and harvested in the exponential (OD_600_ = 1, dark blue) or stationary phase (OD_600_ = 8, light blue) or extracted from G. mellonella cadavers at 1 day (pink), 3 days (dark purple), and 7 days (light purple) post-infection or of an *in vitro* spore preparation in LB medium (gray). Pictures show colonies photographed under a binocular stereo microscope (see details in Materials and Methods), 16 h postplating on LB agar. The scale bar represents 5 mm. Download FIG S5, DOCX file, 3.7 MB. Copyright © 2023 Toukabri et al. 2023 Toukabri et al. https://creativecommons.org/licenses/by/4.0/ This content is distributed under the terms of the Creative Commons Attribution 4.0 International license.

In order to determine if the late-infection stage bacteria were better adapted to a medium closer to the insect cadaver environment, we performed a recovery assay on an Insect agar medium prepared as described in Materials and Methods. Expo cells, Stat cells, spores harvested from 48-h LB cultures at 30°C, and bacteria extracted from insect cadavers at 1, 3, and 7 dpi were plated onto Insect agar plates. Figure 5d shows that there was a drastic drop in the numbers of CFU/mL resulting from the plating of Expo and Stat cells, as well as spores, on Insect agar compared to LB. Less than 0.3% of these bacteria survived on this medium. About 6% of the 1-dpi population was able to grow on this medium compared to LB. In sharp contrast, the number of CFU/mL for 3-and 7-dpi cells was similar on the two media. Taken together, these results indicate that the insect cadaver is a hostile environment for bacteria and that adaptation is required to survive under these conditions.

### Transcriptomic profile of late-infection B. thuringiensis.

**FIG 6:**
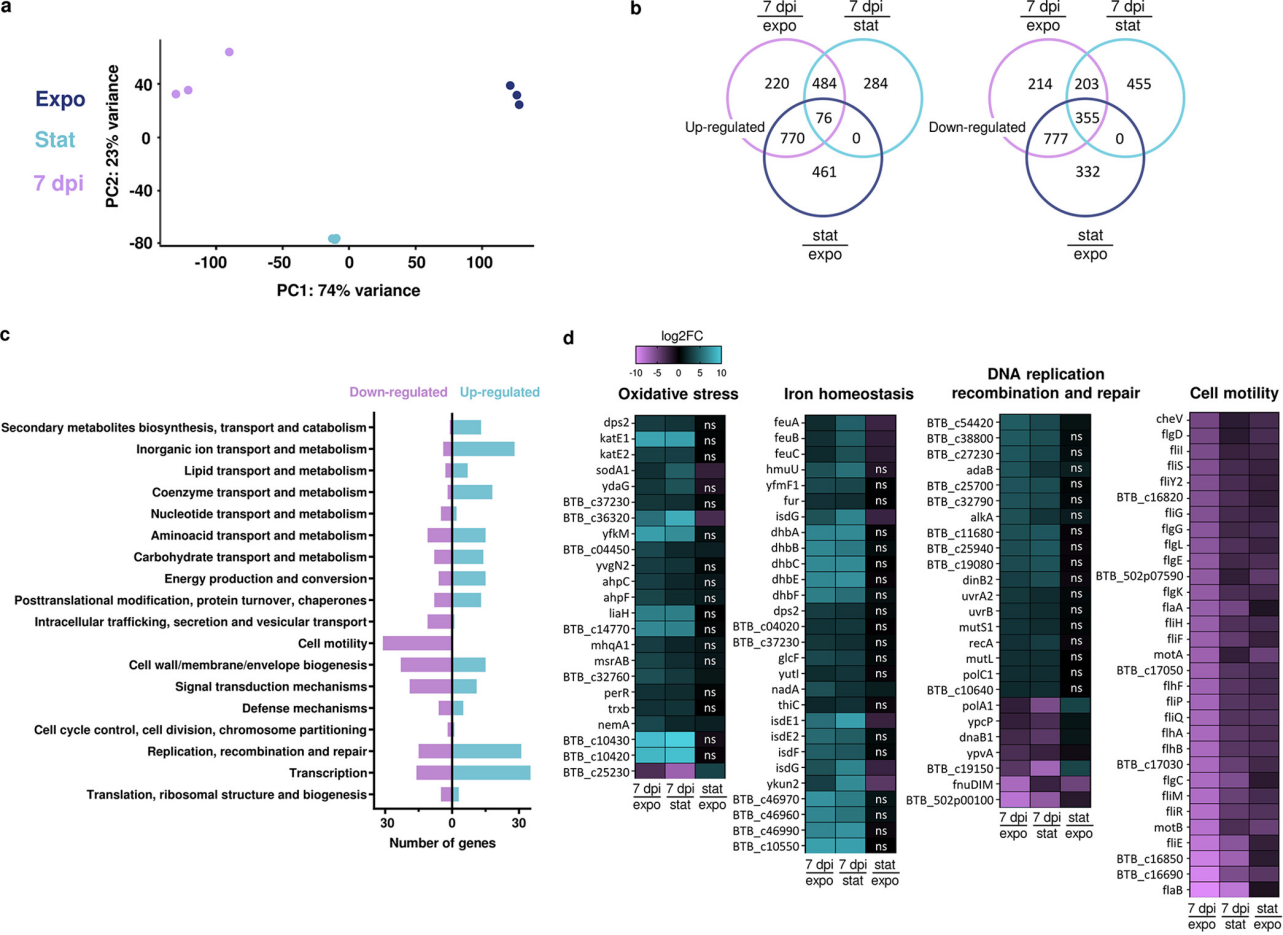
Transcriptomic analysis of bacterial cells extracted from insect cadavers at 7 days post-infection. (a) Principal-component analysis plot of the different samples: exponential-growth-phase cells (dark blue), stationary-growth-phase cells (light blue), and cells extracted from insect cadavers at 7 dpi (purple). Samples were plotted against the first two principal components calculated from the gene expression values. The axis labels indicate the percentage of total variance that is explained by each component. (b) Venn diagrams indicating the number of upregulated and downregulated genes in cells extracted from insect cadavers at 7 dpi compared to exponential-growth-phase cells (purple) or stationary-growth-phase cells (light blue) and in stationary-growth-phase cells compared to exponential-growth-phase cells (dark blue). The genes taken into account present a log_2_ FC of ≤–2 or log_2_ FC of ≥2 and an adjusted *P* value of ≤0.01. (c) COG analysis of genes expressed in cells extracted from insect cadavers at 7 dpi compared to exponential-growth-phase cells and stationary-growth-phase cells. Blue bars indicate upregulated genes with a log_2_ FC of ≥2, and purple bars indicate downregulated genes with a log_2_ FC of ≤–2. The genes considered have an adjusted *P* value of ≤0.01. Genes with unknown functions are not represented. (d) Heatmaps of genes expressed in cells extracted from insect cadavers at 7 dpi compared to exponential-growth-phase cells (left column) or stationary-growth-phase cells (mid column) or in bacteria harvested in the stationary growth phase compared to the exponential growth phase (right column). Selected categories of differentially expressed genes are shown: oxidative stress, iron homeostasis, DNA replication recombination and repair, and cell motility. Purple indicates low expression (log_2_ FC ≤ –2), and blue indicates high expression (log_2_ FC ≥ 2). ns indicates an adjusted *P* value of >0.01.

To determine the expression profile of the Spo^–^/Nec^–^ cells and begin to understand how these bacteria persist in the cadavers, we performed a global *in vivo* transcriptional analysis at a late stage of infection using an RNA sequencing approach. We chose to extract RNA from the total population at 7 dpi, which is a time point that presented very few Nec^+^ bacteria (Fig. 2), and we verified that RNA was only poorly extracted from spores, thus preventing a bias in our analysis (see Materials and Methods). We compared the transcriptome of 7-dpi bacteria to that of Expo and Stat bacteria grown in LB medium. The transcriptome of the Stat and Expo bacteria grown in LB medium was also compared to identify genes differentially expressed in the insect specifically. As presented in Fig. 6a, the principal-component analysis shows that the 7-dpi bacteria differed substantially from both Expo and Stat cells, indicating that these cells are in a different state. The results show that 484 and 203 genes were specifically upregulated and downregulated, respectively, in the 7-dpi cells compared to both Expo and Stat bacteria (Fig. 6b). We found that most of the genes belonging to the NprR regulon were downregulated compared to Stat cells but upregulated compared to Expo cells (Table S2). This is in accordance with the fact that a small proportion of bacteria are Nec^+^ at 7 dpi (Fig. 1), while necrotrophism is repressed in the exponential phase and activated during the stationary phase *in vitro* (20). The data also show that most sporulation and germination genes were not differentially expressed, indicating that these two processes were not triggered in the subpopulation of interest (Table S2).

10.1128/mbio.00371-23.9 TABLE S2 Differential gene expression for necrotrophism, sporulation, and germination genes in insect cadavers at 7 dpi compared to exponential-growth-phase cells (left column) or stationary-growth-phase cells (middle column) or in bacteria harvested in the stationary growth phase compared to the exponential growth phase (right column). Necrotrophism genes were retrieved using the necrotrophism regulon published by Dubois et al. (11); sporulation and germination genes were retrieved from KEGG (22) and the SubtiWiki database (53). Purple indicates low expression (log_2_ FC ≤ –2), blue indicates high expression (log_2_ FC ≥ 2), and gray indicates an adjusted *P* value of > 0,01 or –2 < Log_2_ FC < 2. Download Table S2, XLSX file, 0.03 MB. Copyright © 2023 Toukabri et al. 2023 Toukabri et al. https://creativecommons.org/licenses/by/4.0/ This content is distributed under the terms of the Creative Commons Attribution 4.0 International license.

Most of the upregulated genes in the 7-dpi cells are involved in inorganic ion transport and metabolism, transcription, replication, recombination, and repair, whereas the downregulated genes are mostly involved in cell motility, signal transduction mechanisms, and cell wall/membrane/envelope biogenesis (Fig. 6c), the latter indicating a decrease in the growth process. More than 300 differentially expressed genes were assigned to an unknown function. Differentially expressed genes with the highest or lowest log_2_ fold change (FC) values were found in the oxidative stress, iron homeostasis, DNA replication, recombination and repair, and cell motility categories (Fig. 6d). Interestingly, all motility genes were down, all iron homeostasis genes were up, and all oxidative stress genes were up (except for one), suggesting that these mechanisms are key for-late infection-stage survival.

### Oxidative stress genes are specifically expressed during late infection, in contrast to iron homeostasis genes expressed at all times.

**FIG 7:**
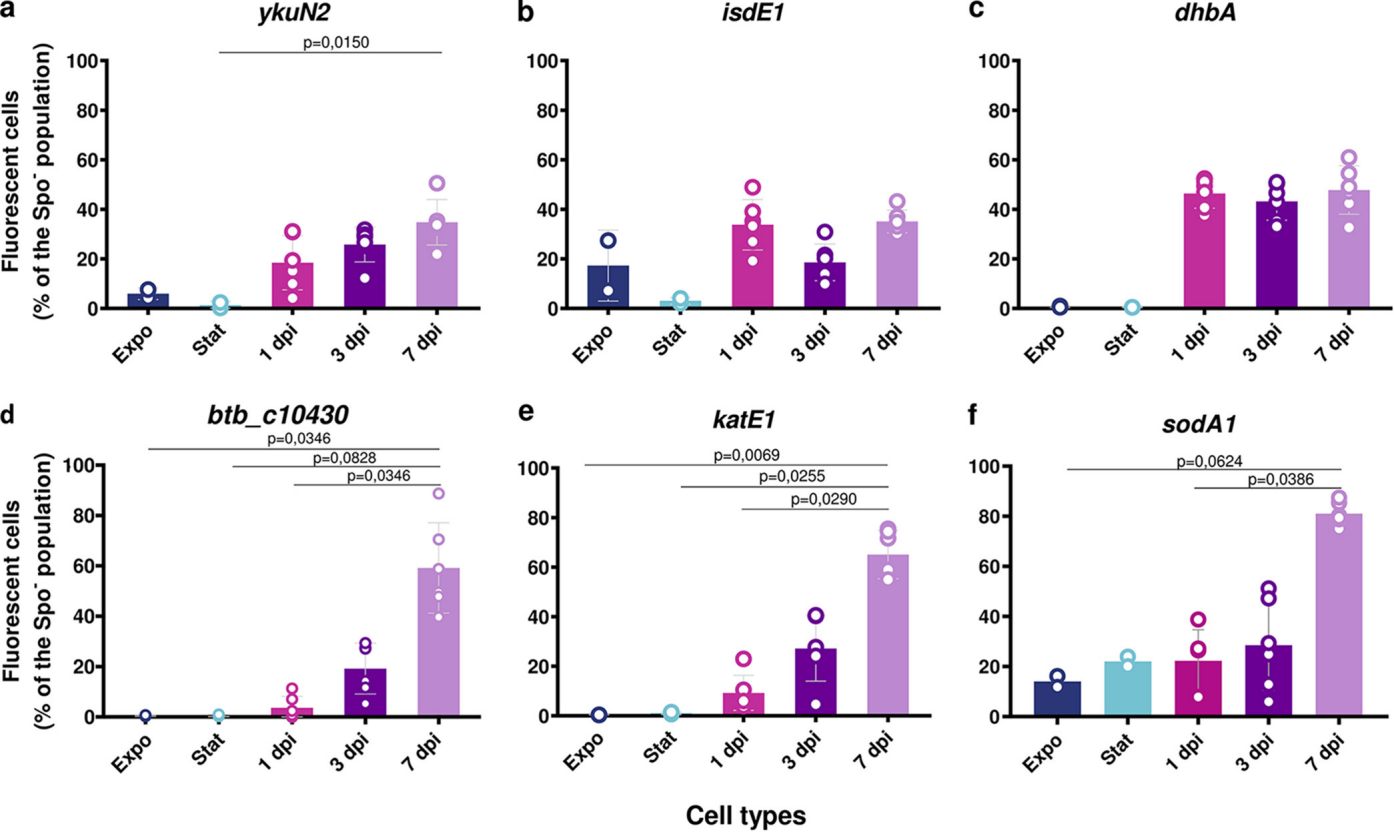
Iron homeostasis and oxidative stress resistance promoter activities during infection. (a to f) Flow cytometry analysis of Bt (pP*ykuN2′gfp_Bte_AAV-*P*spoIIQ′mcherry*) (a), Bt (pP*isdE1′gfp_Bte_AAV-*P*spoIIQ′mcherry*) (b), Bt (pP*dhbA′gfp_Bte_AAV-*P*spoIIQ′mcherry*) (c), Bt (pP*BTB_c10430′gfp_Bte_AAV-*P*spoIIQ′mcherry*) (d), Bt (pP*katE1′gfp_Bte_AAV-*P*spoIIQ′mcherry*) (e), and Bt (pP*sodA1′gfp_Bte_AAV-*P*spoIIQ′mcherry*) (f) cells grown in LB medium and harvested in the exponential (OD_600_ = 1, dark blue) or stationary phase (OD_600_ = 8, light blue) or extracted from G. mellonella cadavers at 1 day (pink), 3 days (dark purple), and 7 days (light purple) p.i. Green fluorescent cells among the nonsporulating bacteria were discriminated in cytograms as described in Materials and Methods. Each symbol represents the data relative to bacteria extracted from one larva. The data are the result of two independent experiments, and the error bars show the standard deviation from the mean. Statistically significant differences are indicated by black bars with the *P* value obtained after a Kruskal-Wallis test followed by Dunn’s multiple-comparison test.

To examine the expression profile of selected genes identified in the RNA sequencing (RNA-Seq) analysis and determine which of these were specific to late-infection stage survival, we constructed strains harboring a transcriptional fusion between the promoter region of the gene of interest and the *gfp_Bte_AAV* reporter gene, associated with the sporulation reporter P*spoIIQ'mcherry*. We chose genes with the highest log_2_ FC values obtained by the differential gene expression analysis in the iron homeostasis and oxidative stress resistance categories. We followed the expression profile of representative genes from these categories, such as *ykuN2* (a flavodoxin that replaces ferredoxin under conditions of iron limitation), *isdE1* (involved in heme scavenging), *dhbA* (the first gene of the *dhb* operon that specifies the biosynthesis of the siderophore bacillibactin involved in the chelation of ferric iron from the surrounding environment), *BTB_c10430* (annotated as *sigX* by KEGG orthology), *katE1* (a catalase and general stress protein), and *sodA1* (a superoxide dismutase and general stress protein) (22). P*ykuN2'gfp_Bte_AAV* and P*isdE1*'*gfp_Bte_AAV* were expressed from the start of the infection, and the percentage of fluorescent cells among the Spo^–^ bacteria was around 30% (Fig. 7a and b). Similarly, P*dhbA'gfp_Bte_AAV* was expressed in approximately 40% of the cells among the Spo^–^ bacteria in the 1-, 3-, and 7-dpi samples (Fig. 7c). For the reporter P*BTB_c10430'gfp_Bte_AAV*, less than 20% of the 1- and 3-dpi Spo^–^ cells were GFP positive. At 7 dpi, around 40% of the Spo^–^ cells produced GFP, indicating a specific activation of *BTB_c10430* in late-infection survival (Fig. 7d). P*katE1'gfp_Bte_AAV* and P*sodA1'gfp_Bte_AAV* were specifically expressed in 7-dpi cells with about 65% of green fluorescent Spo^–^ cells for the former and 80% for the latter (Fig. 7c). None of these reporters were expressed in Expo and Stat cells, except for P*sodA1'gfp_Bte_AAV*. Moreover, only about 20% of the Spo^–^ bacteria expressed those reporters after a 7-day *in vitro* culture (Fig. S6a). Altogether, these results indicate that iron homeostasis is important from the start of the infection, whereas an oxidative stress response is mounted as the bacteria spend more time in the cadaver environment.

10.1128/mbio.00371-23.7 FIG S6 Oxidative stress resistance promoter activities in cells extracted from 7 days postinoculation LB cultures and free radical detection in LB media. (a) Flow cytometry analysis of Bt (pP*katE1′gfp_Bte_AAV-*P*spoIIQ′mcherry*) and Bt (pP*sodA1′gfp_Bte_AAV-*P*spoIIQ′mcherry*) cells grown in LB medium for 7 days. Green fluorescent cells among the nonsporulating bacteria were discriminated in cytograms as described in Materials and Methods. (b) Extracellular ROS/RNS assay. Quantification of total free radicals in LB from 7-day-postinoculation cultures (pink) and LB medium (purple) as described in Materials and Methods. Each symbol represents the data relative to bacteria harvested from one culture or one LB sample. The data are the results of three independent experiments, and the error bars show the standard deviation from the mean. *, 2 values were below the detection threshold. Download FIG S6, DOCX file, 0.04 MB. Copyright © 2023 Toukabri et al. 2023 Toukabri et al. https://creativecommons.org/licenses/by/4.0/ This content is distributed under the terms of the Creative Commons Attribution 4.0 International license.

### Free radical level increases in late-infection stages in G. mellonella cadavers and B. thuringiensis cells.

**FIG 8:**
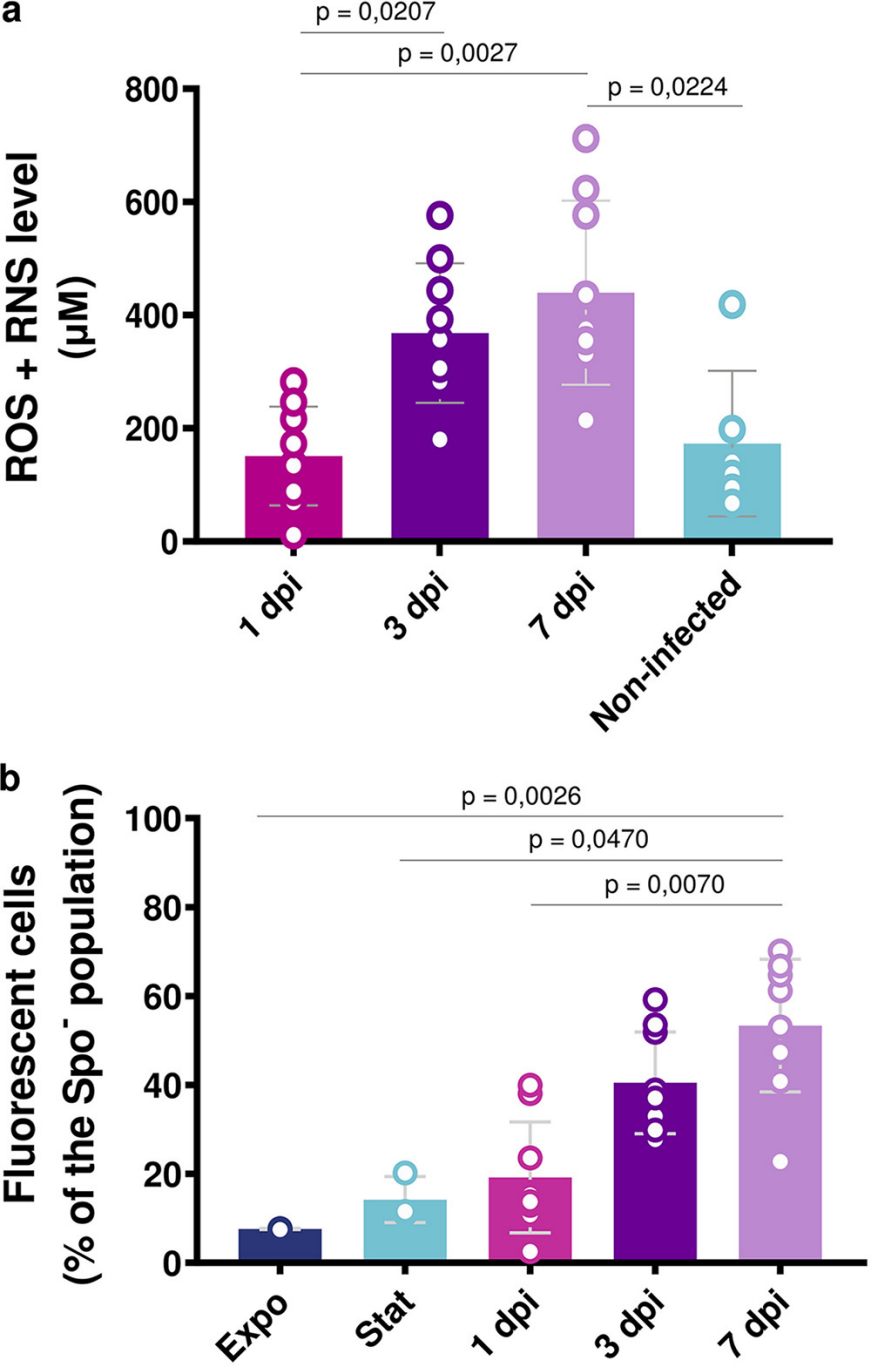
Free radical detection during infection. (a) Extracellular ROS/RNS assay. Quantification of total free radicals in G. mellonella supernatants at 1 day (pink), 3 day (dark purple), and 7 days (light purple) post-infection and in noninfected larvae supernatants killed by immersion in liquid nitrogen (light blue), as described in Materials and Methods. (b). Intracellular ROS assay. Flow cytometry analysis of Bt (pP*spoIIQ′mcherry*) cells grown in LB medium and harvested in the exponential (OD_600_ = 1, dark blue) or stationary phase (OD_600_ = 8, light blue) or extracted from G. mellonella cadavers at 1 day (pink), 3 days (dark purple), and 7 days (light purple) post-infection. The samples were incubated for 30 min in the dark at 37°C with 10 μM DCFDA before being subjected to analysis. The percentage of marked cells among nonsporulating bacteria discriminated in cytograms (as described in Materials and Methods) is presented as a function of time. Unmarked bacteria were used as controls. Each symbol represents the data relative to one larva or one culture supernatant (a) or bacteria extracted from one larva or one culture (b), with the bars representing the means of these data. The data are the result of three independent experiments, and the error bars show the standard deviation from the mean. Statistically significant differences are indicated by black bars with the *P* value obtained after a Kruskal-Wallis test followed by Dunn’s multiple-comparison test.

In order to understand the environmental conditions encountered by bacteria in the insect cadaver and determine if oxidative stress is generated in the late infection stages compared to the early stages, the level of total free radicals (reactive oxygen species, or ROS, and reactive nitrogen species, or RNS) was compared in insect cadavers at 1, 3, and 7 dpi. We also measured the level of total ROS/RNS in noninfected larvae, 7 days post-killing by immersion in liquid nitrogen, to examine the effect of death by septicemia on this response. The free radical levels were measured in the supernatants of crushed larvae as described in Materials and Methods. The results show that total ROS/RNS levels reached a mean value of approximately 400 μM in 3- and 7-dpi cadavers, compared to less than 200 μM in 1-dpi and noninfected cadavers (Fig. 8a). Total free radicals were also measured in LB medium and in the supernatant of 7-day-postinoculation B. thuringiensis cultures, and their levels were below 50 μM (Fig. S6b). These results indicate that the late-infection stage cadaver constitutes an oxidative environment and suggest that this feature is linked to death by septicemia.

To assess the oxidative status of B. thuringiensis cells during infection, we measured the intracellular ROS level of strain Bt (pP*spoIIQ′mcherry*) cultured in LB and harvested in the exponential growth phase (Expo) and stationary growth phase (Stat) or extracted from insect cadavers at 1, 3, and 7 dpi, using the molecular dye DCFDA as described in Materials and Methods. This dye permeates the cells and ultimately becomes fluorescent in the presence of ROS. The bacteria were analyzed by flow cytometry (Fig. 8b) to quantify the fluorescently marked Spo^–^ cells. The results show that about 50% and 40% of 7-dpi and 3-dpi Spo^–^ cells were stained, respectively, whereas less than 20% of Expo, Stat, and 1-dpi cells were fluorescently marked. These results show that the number of ROS-producing bacteria increases with the infection time and is more elevated than for *in vitro*-grown cells. Altogether, these data are in agreement with the RNA-Seq and single-cell fluorescent promoter analyses (Fig. 6d and 7d to f) that show that late-infection stage bacteria mount an oxidative stress response.

## DISCUSSION

Our study reports the characterization of a physiological state that creates for the sporulating entomopathogenic Gram-positive bacterium B. thuringiensis specific properties allowing its persistence in the host environment in a nonsporulated form. We analyzed the phenotypic properties and transcription profile of the Spo^–^ B. thuringiensis cells in the context of an infection in G. mellonella insect larvae. These larvae are natural hosts for the B. thuringiensis species, but they are also widely used as an alternative to mammalian models of infection to study bacterial virulence and host colonization (23, 24).

The RNA-Seq analysis showed that the motility genes were downregulated in 7-dpi bacteria compared to *in vitro*-grown cells and that the motility repressor-encoding gene *mogR* (25) was upregulated in 7-dpi bacteria compared to exponentially growing cells. Similarly, the biofilm-associated genes *calY* and *tasA* and the *eps2* locus (26, 27) were upregulated compared to exponentially growing cells. These data suggest that the late-infection stage bacteria form a biofilm which might confer them an advantage for long-term survival considering the resistance properties of these structures (28). The most represented categories among the upregulated transcripts in 7-dpi bacteria compared to *in vitro*-grown cells are the oxidative stress response, DNA recombination, replication, and repair, in addition to iron homeostasis. The expression of uptake systems for iron, as well as biosynthesis and uptake of the endogenous siderophore bacillibactin, was shown to be activated by iron limitation in B. cereus (29). During an infection, the host is considered an iron-depleted environment, and bacteria need to deploy strategies to scavenge this element, such as the activation of the Fur regulon (30, 31). This is consistent with the activation of the iron homeostasis genes *dhbA* and *isdE1*, belonging to this regulon, during all stages of the infection. Iron is essential for the function of various proteins, and iron homeostasis was previously reported as being important for B. cereus pathogenesis in the first stages of G. mellonella infection by oral gavage (32). However, this metal is also a potential hazard in combination with compounds such as H_2_O_2_ because the reaction between the two products generates reactive oxygen species (ROS) that can be harmful by damaging cellular components such as DNA or proteins with [Fe-S] clusters (33). Several studies have linked iron homeostasis and DNA repair to a response to oxidative stress and showed the upregulation of these gene categories upon H_2_O_2_ exposure (34, 35), and it was recently reported that H_2_O_2_ was produced in the hemolymph of G. mellonella during Salmonella enterica infection (36). Moreover, the melanization defense reaction that occurs after the insect infection liberates ROS and might last for several days (37–39). Aerobic metabolism also generates endogenous oxidative stress if O_2_ is not completely reduced during respiration, and a secondary oxidative stress response can also be triggered when *Bacillus* bacteria encounter unfavorable conditions in an aerobic environment (40). We have shown that the level of free radicals increases in the insect cadaver as it ages and that the number of ROS-producing bacteria also increases with time. All these stresses are counteracted by the action of proteins such as catalases (Kat), superoxide dismutases (SodA), and DNA-binding ferroxidases (Dps) (41, 42), all of which are overexpressed in 7-dpi bacteria. RNA-Seq analysis also revealed that the BTB_c10430 locus, annotated as encoding the SigX protein (22), is specifically upregulated in 7-dpi bacteria. SigX belongs to the extracytoplasmic function (ECF) sigma factor family that helps maintain cell envelope homeostasis and activate resistance to agents that can compromise the integrity of the envelope, the first defense against environmental threats (43). A B. subtilis *sigX* deletion mutant was shown to be sensitive to oxidative stress (44). Further investigations should examine the involvement of this regulatory protein in the maintenance of B. thuringiensis in insect cadavers. It would also be informative to determine if the oxidative stress response triggered in the late-infection stage bacteria is due to the cadaver environment, to excessive iron uptake, or to a combination of the two and to understand the role of the oxidative stress response in the resistance of B. thuringiensis during long-term infection. Our results suggest that the increase in the ROS/RNS level is linked to the infection since this level is lower in G. mellonella cadavers that did not die from septicemia. This is potentially due to the melanization reaction that did not occur in the latter to the same extent as in the infected larvae (data not shown).

B. thuringiensis is able to overcome the immune defenses of G. mellonella, to multiply, and to completely invade its host in less than 24 hpi (11, 45, 46) (this study and our unpublished data). It was shown that the bacteria engage in necrotrophism after the death of the larva by activating the NprR regulon that includes genes encoding chitinases, proteases, and oligopeptide permeases (11, 15). Activation of this regulon seemed to last throughout the duration of the infection (15), and an *nprR* deletion mutant was unable to survive in the cadaver, suggesting that the regulon was required for long-term survival. However, we showed here that necrotrophism is a transient state limited to the first stages of survival in the cadaver. We propose that this state allows B. thuringiensis to scavenge the nutrients present in the cadaver shortly after the insect death, and then the bacteria enter different pathways, potentially triggered by a stress signal such as nutrient depletion, that allow for their persistence in the host. A subpopulation enters the differentiation pathway, leading to sporulation, while another engages in a metabolic slowdown that is accentuated with the increase of exposure time to the cadaver environment. This subpopulation also showed resistance to the cadaver environment, which proved hostile for *in vitro*-grown bacteria as well as *in vitro*-generated spores.

Altogether, these results show that nonsporulated late-infection stage B. thuringiensis bacteria are resilient cells able to resist in a harsh environment and suggest that this adaptation relies at least in part on the activation of stress resistance genes as well as on entering a metabolic slowdown. The fitness advantages conferred by the phenotypic heterogeneity of the B. thuringiensis population during infection and, in particular, by the coexistence of spores and of the nonsporulated form able to survive for a prolonged period in a host cadaver, still need to be elucidated. Studying the mechanisms that govern this state will provide valuable fundamental knowledge about the life cycle of these bacteria and might lead to the development of new strategies to combat sporulating pathogenic species.

## MATERIALS AND METHODS

### Bacterial strains and growth conditions.

The acrystalliferous Bacillus thuringiensis 407 Cry^−^ strain (B. thuringiensis 407^−^) (47) was used as the parental strain to create all the strains used in this study. Escherichia coli strain DH5α (48) was used as the host strain for plasmid construction. E. coli strain ET12567 (49) was used to prepare DNA prior to electroporation in B. thuringiensis. Cells were grown with shaking at 30°C or 37°C in LB medium (1% tryptone, 0.5% yeast extract, 1% NaCl) or HCT medium (0.7% casein hydrolysate, 0.5% tryptone, 0.68% KH2PO4, 0.012% MgSO4 · 7H2O, 0.00022% MnSO4 · 4H2O, 0.0014% ZnSO4 · 7H2O, 0.008% ferric ammonium citrate, 0.018% CaCl2 · 4H2O, pH 7.2). The bacteria were stored at −80°C in LB containing 15% glycerol.

When required, the medium was supplemented with antibiotics at the following concentrations: 100 μg/mL ampicillin (for E. coli) and 10 μg/mL erythromycin (for B. thuringiensis).

### Plasmid and strain construction.

DNA manipulations are detailed in the Text S1. All the plasmids, strains, and oligonucleotide primers used in this study are listed in Tables S1a to c.

10.1128/mbio.00371-23.1 TEXT S1 Additional experimental details about DNA manipulation, flow cytometry analysis, RNA extraction, RNA-Seq analysis, and ROS/RNS assays. Download Text S1, DOCX file, 0.04 MB. Copyright © 2023 Toukabri et al. 2023 Toukabri et al. https://creativecommons.org/licenses/by/4.0/ This content is distributed under the terms of the Creative Commons Attribution 4.0 International license.

10.1128/mbio.00371-23.8 TABLE S1 **(a to c)** Plasmids (a), strains (b), and oligonucleotides primers (c) used in this study. Download Table S1, DOCX file, 0.04 MB. Copyright © 2023 Toukabri et al. 2023 Toukabri et al. https://creativecommons.org/licenses/by/4.0/ This content is distributed under the terms of the Creative Commons Attribution 4.0 International license.

### Infection of Galleria mellonella.

Intrahemocoelic injection experiments with Galleria mellonella were carried out as described previously (14). Briefly, last-instar larvae were infected by intrahemocoelic injections with 2.10^4^ vegetative cells in exponential phase (optical density at 600 nm [OD_600_], 1). Inocula were counted after plating onto LB agar medium. Infected larvae were incubated at 30°C under a Nikon CoolPix P1 device on time-lapse photography mode, and pictures were taken every 10 min to determine the time of death for each larva. Larvae that died within the same hour were used for each time point. A larva was considered dead at the time its movements stopped if its body melanized afterwards (see Fig. S1).

### Extraction of B. thuringiensis from G. mellonella cadavers.

The method of isolation of bacteria from insect cadavers was adapted from Verplaetse et al. (15). Cadavers were crushed with a 1-mL tip in a 1.5-mL Eppendorf tube. Then, 1 mL of saline was added, and the tube was vortexed. Next, 750 μL of the suspension was transferred to a new 1.5-mL tube and centrifuged for 45 s at 13,000 rpm. The pellet was resuspended in 750 μL of saline and loaded in a 1-mL syringe fitted with a cotton filter in order to isolate bacterial cells from cadaver debris. The filtrate was then centrifuged for 30 s at 13,000 rpm, fixed in 4% formaldehyde in saline for 15 min, washed in saline, resuspended in GTE buffer (50), and kept at 4°C until flow cytometric or microscopic analysis.

### Flow cytometric analysis.

Bacteria were diluted in filtered saline, and fluorescent events were measured on a CyFlow Space cytometer (Sysmex Partec, France). Specifications of the apparatus and flow cytometric analyses are described in Text S1.

### Microscopy.

Bacteria were observed with an AxioObserver.Z1 Zeiss inverted fluorescence microscope with a Zeiss AxioCam MRm digital camera and with Zeiss fluorescence filters. GFP, 5(6)-CFDA, DiBAC4(3), and Sytox Green were imaged using the 38 HE filter (excitation: BP 470/40, beam splitter: FT 495, emission: 525/50), and mCherry was imaged using the 45 HE filter (excitation: BP 590/20, beam splitter: FT 605, emission: 620/14). Images were processed using the ZEN software package (Zeiss).

### Sporulation assay.

The sporulated population was assessed by plating and by flow cytometry. Each larva cadaver was ground in 3 mL of saline on ice using a T10 Ultra-Turaxx apparatus. The volume was then adjusted to 10 mL. To determine the number of total cells and the number of heat-resistant spores, the bacteria were enumerated by plating on LB agar plates following serial dilutions before and after heat treatment at 80°C for 12 min. For flow cytometry, fixed bacteria (as described above) harboring the red fluorescent sporulation reporter P*spoIIQ'mcherry* were analyzed. Red fluorescent events were counted among the total cell count to calculate the percentage of sporulation.

### Induction of GFP expression.

Bacteria were extracted from insect cadavers as described above or were harvested from HCT cultures at 30°C at an OD_600_ of 1 (exponential phase of growth) and OD_600_ of 6 (stationary phase of growth) via centrifugation at 13,000 rpm for 30 s, washed once in saline, and resuspended in conditioned HCT medium (obtained after filtration of a 10-h culture of B. thuringiensis 407^–^ in HCT) to preserve bacteria while preventing cell growth. Half of the suspension was supplemented with xylose at a 25-mM final concentration. For each sample, an aliquot was fixed as described above at the time of xylose addition and after 1 and 2 h of incubation with shaking at 30°C. The samples were kept at 4°C until flow cytometric or microscopic analysis.

### Growth recovery assessment of the bacteria extracted from insect cadavers.

Culturability was assessed by plating the bacteria on two different media, LB agar and Insect agar, and by time-lapse microscopy. The bacteria were extracted from insect cadavers as described above or were harvested from *in vitro* cultures in LB at 30°C at an OD_600_ of 1 (exponential phase) and an OD_600_ of 8 (stationary phase) and from a 48-h culture (composed of about 65% of spores) via centrifugation at 13,000 rpm for 30 s, washed once in saline, serial diluted, and plated onto LB plates containing erythromycin. Then, 16 h after incubation at room temperature, colonies were photographed with a Canon Eos 750D camera under a binocular stereo microscope Leica MZFLIII. Colony area was determined using ImageJ software (51).

To prepare Insect agar plates, 12 larva cadavers were harvested at 7 dpi and crushed in 50 mL saline and then filtered as described above. Agar prepared in saline was then added at a final concentration of 1.5%. The bacteria extracted from insect larvae or harvested from LB cultures, in the same manner as described above, were serial diluted and plated onto LB plates supplemented with erythromycin and Insect agar plates supplemented with erythromycin. Colonies were counted 16 h after incubation at room temperature for LB plates and after 4 days for Insect agar plates.

For time-lapse microscopy, medium pads were prepared with LB supplemented with 1% agarose to fill a 125-μL Gene Frame (Thermo Fisher Scientific, Waltham, MA, USA). After polymerization, 2-mm-wide strips were cut, and three strips were placed in a new Gene Frame. Bacteria were extracted from insect larvae or harvested from LB cultures as described above and washed in saline. Then, 2 μL of cells was pipetted onto the strips before the frame was sealed with a coverslip. B. thuringiensis development was monitored at 30°C in a temperature-controlled chamber with the microscope described above. Phase-contrast and fluorescence images were taken every hour for 5 h. Only bacteria extracted from insect cadavers at 0 h were assigned to a category (elongation, division, lysis, inactivity). Newly formed daughter cells were excluded from the analysis.

### Molecular dye staining.

Bacterial cells were harvested from LB cultures or insect cadavers as described above and resuspended in 1 mL of saline. Samples were diluted to 10^7^ cells/mL in filtered saline supplemented or not with one of the dyes. 5(6)-CFDA (5-(and-6)-carboxyfluorescein diacetate) (Biotium, Fremont, CA, USA), DiBAC4(3) (Bis-(1,3-dibutylbarbituric Acid) trimethine oxonol) (Sigma-Aldrich, Saint-Louis, MO, USA), and Sytox Green (Invitrogen, Eugene, OR, USA) were suspended in dimethyl sulfoxide (DMSO), and DCFDA (Dichlorofluorescin diacetate) (Abcam, USA) was suspended in the manufacturer’s buffer. The dyes were used at a final concentration of 10 μM (DCFDA), 5 μM [5(6)-CFDA], or 0.5 μM [DiBAC4(3) and Sytox Green]. Bacteria were incubated at room temperature for 15 min in the dark except for DCFDA, which required an incubation at 37°C for 30 min. Stained samples were washed once in saline before flow cytometric or microscopic analysis. Unstained cells (for all dyes) and cells heat-shocked at 90°C for 15 min [for 5(6)-CFDA, DiBAC4(3), and Sytox Green] were used as controls.

### RNA-Seq analysis.

RNA was extracted from shaken liquid cultures and from dead larvae at 7 dpi as described in the Text S1. Nanodrop and Bioanalyzer instruments were used for quantity and quality controls (6.8 ≤ RINs (RNA Integrity Number) ≤ 10). Disrupting cells broke Spo^–^ bacteria and left spores intact. Moreover, extraction of RNA from spore preparations using our protocol resulted in a very low yield of poor quality. However, we cannot exclude minute contamination with RNA from spores.

Library preparation including rRNA depletion (RiboZero) and sequencing was performed using the I2BC platform (Gif-sur-Yvette, France). Sequencing was conducted on an Illumina NextSeq machine using the NextSeq 550/500 midoutput kit v2 to generate paired-end reads (2 × 75).

Data processing was performed with the I2BC platform, including the following steps: demultiplexing (with bcl2fastq2-2.18.12), adapter trimming (Cutadapt v1.15), quality control (FastQC v0.11.5), mapping (Burrows-Wheeler Algorithm [BWA] v0.6.2-r126) against B. thuringiensis strain 407. This generated 10 to 20 million paired-end reads per sample and counts for 6,530 genes. Differentially expressed genes (DEGs) were determined using the R package DESeq2 v1.30.1 to estimate *P* values and log_2_ fold changes. DEGs with an adjusted *P* value (padj) of ≤10^−2^ and log_2_ FC of ≤–2 or log_2_ FC of ≥2 were used for further analyses. Gene expression data are being deposited at the public repository Gene Expression Omnibus (accession number GSE206636).

Using the functional reannotation database available in the laboratory (see details in supplemental materials) of the B. thuringiensis 407 genome (chromosome: International Nucleotide Sequence Database Collaboration [INSDC] CP003889.1, plasmids: INSDC CP003890.1 to CP003898.1), GO terms, COG letters, and B. subtilis homologous genes were assigned to our data set with Microsoft SQL server 2019. Venn diagrams were constructed with Venny v2.1.0 (52), (https://bioinfogp.cnb.csic.es/tools/venny/index.html). Using SubtiWiki (53), the KEGG orthology database (22), and COG letters, we retrieved genes from these functional categories: oxidative stress, iron homeostasis, DNA recombination and repair, necrotrophism, sporulation, and germination (see Table S2 for necrotrophism, sporulation, and germination). Histograms and heatmaps of DEGs according to these functional categories were then constructed using Prism v8 software (GraphPad).

### ROS/RNS assay.

Total free radicals (reactive oxygen species, or ROS, and reactive nitrogen species, or RNS) were quantified in G. mellonella at 1, 3, and 7 days post-infection and in noninfected larvae killed by immersion in liquid nitrogen, using the OxiSelect *in vitro* ROS/RNS assay kit (Cell Biolabs, USA) according to the manufacturer’s instructions. Closed Eppendorf tubes containing noninfected larvae were placed in a cryobucket and immersed in liquid nitrogen. For the other conditions, larvae were infected by B. thuringiensis as described above. Noninfected larvae, or infected larvae collected at 1, 3, and 7 dpi, were crushed in saline and centrifuged at 13,000 rpm for 45 s. All supernatants were collected, diluted 10-fold, and incubated with the OxiSelect kit components in a 96-well black-bottom plate for 30 min at room temperature. Fluorescence was then measured, and the results were obtained from the linear regression equation of a predetermined DCF (Dichlorofluorescein) standard curve. Measurements were performed on an Infinite M200 Pro microplate spectrofluorometer (Tecan, USA).

### Statistical analysis.

The data were analyzed using Prism v8 software (GraphPad).
