# Supplementary material for: A Sporulation-Independent Way of Life for Bacillus thuringiensis in the Late Stages of an Infection
Source: mBio. 2023 Apr 27;14(3):e00371-23. doi: 10.1128/mbio.00371-23 (PMC10294645; doi:10.1128/mbio.00371-23)
Supplement: FIG S1 [file mbio.00371-23-s0004.docx]

**Figure S1**


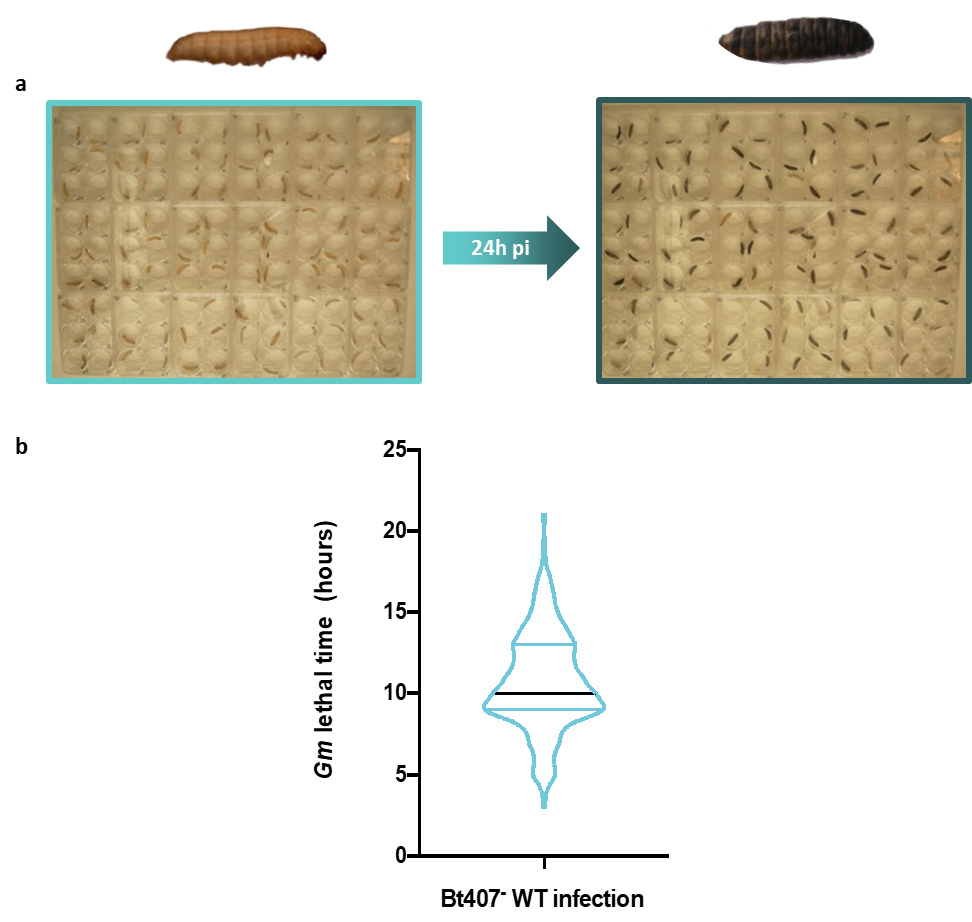


***G. mellonella* lethal time (hours)**

***B. thuringiensis* infection**

**24 hpi**

**Figure S1. Time-lapse photography set-up to determine *G. mellonella* lethal time. a.** First picture shows infected larvae incubated in 6-well plates at 30°C under a Nikon CoolPix P1 camera on time-lapse photography mode to determine the time of death for each larva. Melanization as shown by the second picture at 24 hpi and absence of movement are required to consider a larva as dead. **b.** *G. mellonella* lethal time after infection with *B. thuringiensis* strain 407. A picture was taken every 10 min with the time-lapse photography set-up and lethal time post-infection is reported. Black line indicates the median, blue lines indicate the first and last quartile, n>150.
