# Supplementary material for: A Sporulation-Independent Way of Life for Bacillus thuringiensis in the Late Stages of an Infection
Source: mBio. 2023 Apr 27;14(3):e00371-23. doi: 10.1128/mbio.00371-23 (PMC10294645; doi:10.1128/mbio.00371-23)
Supplement: FIG S2 [file mbio.00371-23-s0005.docx]

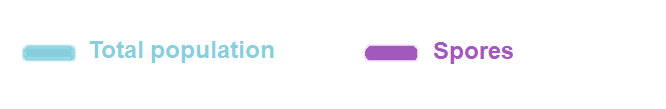
**
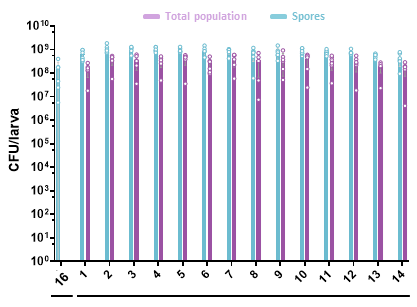
Figure S2**

**Hours** **Days**

**Time post-infection**

**Figure S2. Monitoring of *B. thuringiensis* survival during long-term infection.** The total bacterial population (purple) and spores (blue) were numerated daily for 14 days after intrahemocoelic infection of *G. mellonella* with Bt (pP*nprA’gfp_Bte_AAV*-pP*spoIIQ’mCherry*). Larvae were crushed at the time points indicated and serial dilutions of the homogenate were directly plated onto LB agar for total population numeration. The bacterial suspensions were heated at 80°C during 12 minutes and plated onto LB agar to count heat-resistant spores. Each symbol represents bacteria extracted from one larva. 3 larvae were used for each experiment and time point. The data are the result of two independent experiments and the error bars show the standard deviation from the mean.
