# Supplementary material for: A Sporulation-Independent Way of Life for Bacillus thuringiensis in the Late Stages of an Infection
Source: mBio. 2023 Apr 27;14(3):e00371-23. doi: 10.1128/mbio.00371-23 (PMC10294645; doi:10.1128/mbio.00371-23)
Supplement: FIG S3 [file mbio.00371-23-s0006.docx]

**Figure S3**

**
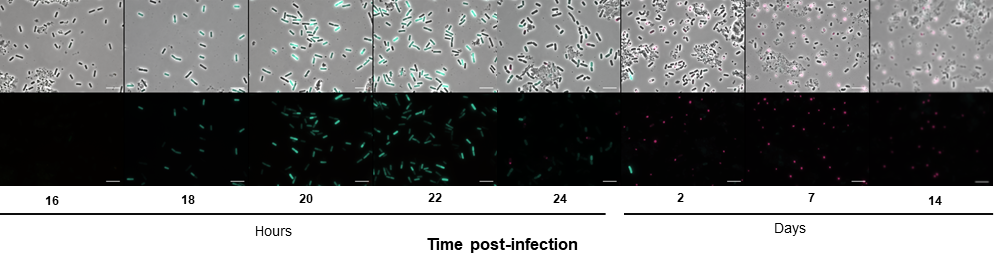
**

**Figure S3. Microscopy observations of the necrotrophism and sporulation promoters activity in bacterial cells during long-term infection.** Bacteria were analyzed by fluorescence microscopy at the time points indicated. Upper panels merge between the phase contrast and epifluorescence images channels; lower panels, epifluorescence images. Cells were false colored in green for Nec^+^ cells and pink for Spo^+^ cells. The scale bars represents 10 μm.
