# Supplementary material for: A Sporulation-Independent Way of Life for Bacillus thuringiensis in the Late Stages of an Infection
Source: mBio. 2023 Apr 27;14(3):e00371-23. doi: 10.1128/mbio.00371-23 (PMC10294645; doi:10.1128/mbio.00371-23)
Supplement: FIG S4 [file mbio.00371-23-s0007.docx]

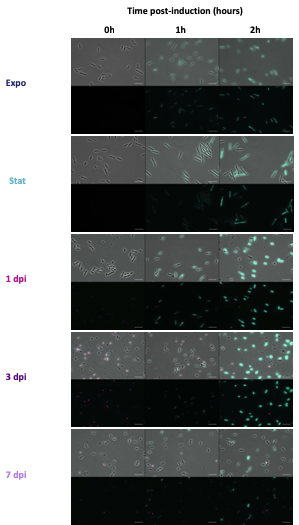
**Figure S4**

**Figure S4. Induction of *gfp* expression in non-sporulated bacteria**. Fluorescence microscopy images at the time after induction indicated above the pictures. Lower panels, epifluorescence images; upper panels, merge between the two channels. Cells were false colored in green for Gfp-expressing cells and pink for Spo^+^ cells. The scale bar represents 10 μm. These results are representative of three independent experiments.
