# Supplementary material for: A Sporulation-Independent Way of Life for Bacillus thuringiensis in the Late Stages of an Infection
Source: mBio. 2023 Apr 27;14(3):e00371-23. doi: 10.1128/mbio.00371-23 (PMC10294645; doi:10.1128/mbio.00371-23)
Supplement: FIG S5 [file mbio.00371-23-s0008.docx]

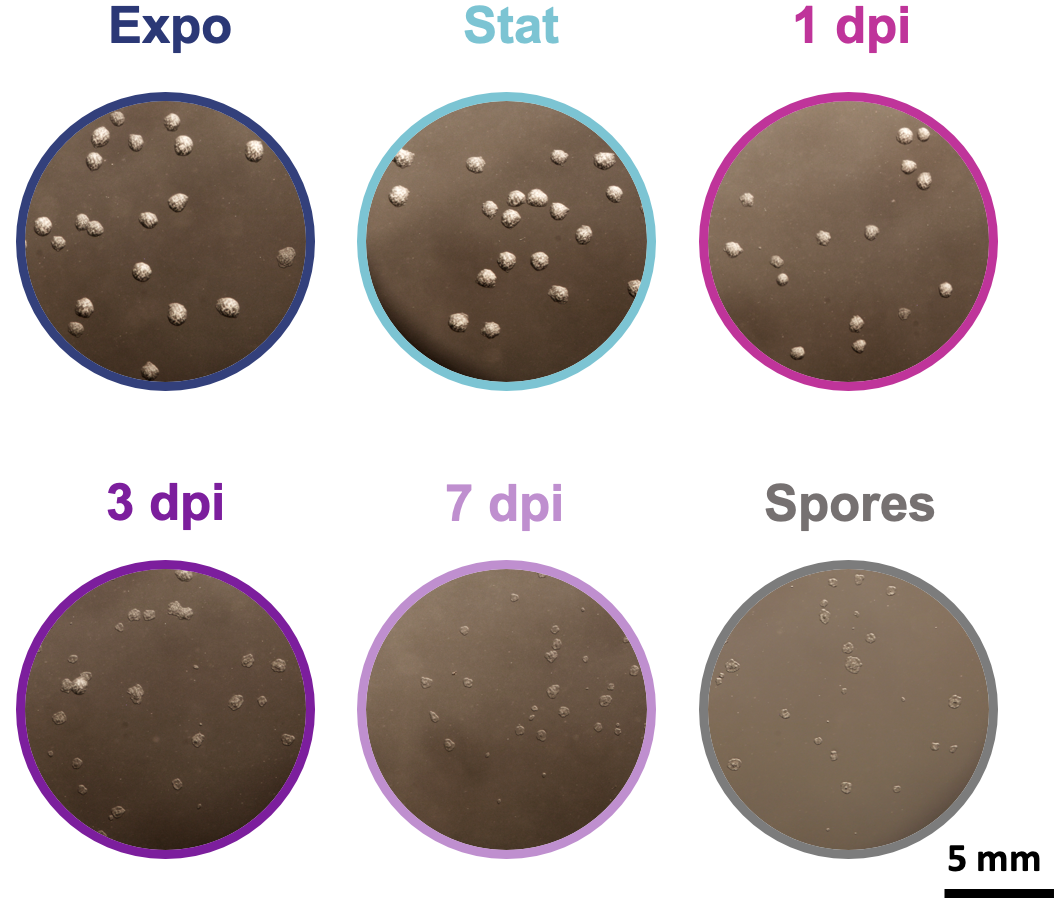


**Figure S5. Recovery assessment on LB agar plates**. Plating of Bt (pP*spoIIQ’mcherry*) cells grown in LB medium and harvested in exponential (OD_600_ = 1, dark blue) or stationary phase (OD_600_ = 8, light blue), or extracted from *G. mellonella* cadavers at 1 day (pink), 3 (dark purple) and 7 days (light purple) post-infection, or of an *in vitro* spore preparation in LB medium (grey). Pictures show colonies photographed under a binocular stereo microscope (see details in the Materials and Methods section), 16 hours post-plating on LB agar. The scale bar represents 5 mm.
