# Supplementary material for: A Sporulation-Independent Way of Life for Bacillus thuringiensis in the Late Stages of an Infection
Source: mBio. 2023 Apr 27;14(3):e00371-23. doi: 10.1128/mbio.00371-23 (PMC10294645; doi:10.1128/mbio.00371-23)
Supplement: FIG S6 [file mbio.00371-23-s0009.docx]

**ROS + RNS level**

**(μM)**

*****

**b**

**a**

**Figure S6. Oxidative stress resistance promoter activities in cells extracted from 7-days post-inoculation LB cultures and free radical detection in LB media.** **a.** Flow cytometry analysis of Bt (pP*katE1’gfp_Bte_AAV-*P*spoIIQ’mcherry*) and Bt (pP*sodA1’gfp_Bte_AAV-*P*spoIIQ’mcherry*) cells grown in LB medium during 7 days. Green-fluorescent cells among the non-sporulating bacteria were discriminated in cytograms as described in the Materials and Methods section **b.** Extracellular ROS/RNS assay. Quantification of total free radicals in LB from 7 days post-inoculation cultures (pink) and LB media (purple) as described in the Material and Methods section. Each symbol represents the data relative to bacteria harvested from one culture or one LB sample. The data are the result of three independent experiments and the error bars show the standard deviation from the mean. * indicates that 2 values were below the detection threshold.
