# Supplementary material for: A Sporulation-Independent Way of Life for Bacillus thuringiensis in the Late Stages of an Infection
Source: mBio. 2023 Apr 27;14(3):e00371-23. doi: 10.1128/mbio.00371-23 (PMC10294645; doi:10.1128/mbio.00371-23)
Supplement: TEXT S1 [file mbio.00371-23-s0001.docx]

**A sporulation-independent way of life for *Bacillus thuringiensis***

**in the late stages of an infection**

Hasna Toukabri, Didier Lereclus and Leyla Slamti*

Micalis Institute, INRAE, AgroParisTech, Université Paris-Saclay, 78350 Jouy-en-Josas, France

*Corresponding author: leyla.slamti@inrae.fr

The authors declare that there are no competing interests in relation to the work described.

**MATERIALS AND METHODS**

**DNA manipulations**

Plasmid DNA was extracted from *E. coli* by a standard alkaline lysis procedure, using a Promega kit (Promega,Madison, Wisconsin USA). Restriction enzymes, T4 DNA ligase, Standard Taq DNA polymerase and Phusion high-fidelity DNA polymerase were purchased from New England Biolabs (Ipswich, MA, USA) and used as recommended by the manufacturer. The oligonucleotide primers (Table S3) used for PCR amplification were synthesized by Eurofins Genomics (Nantes, France). PCR was performed with a 2720 Thermak cycler (Applied Biosystems). All constructs were systematically verified by PCR followed by sequencing of the region of interest. Nucleotide sequences were determined by Eurofins Genomics (Köln, Germany).

**Flow cytometric analysis**

For GFP-, 5(6)-CFDA-, DiBAC4(3)-, Sytox Green- and DCFDA- based fluorescence, a solid blue-laser emitting at 488 nm was used, combined to a 500-nm long pass dichroic mirror and a 527-nm band pass filter (512–542) (FL1 Channel). For mCherry-based fluorescence, a solid yellow-laser emitting at 561 nm was used, combined to a 610-nm long-pass filter (FL4 channel). The analyses were performed using logarithmic gains and detector settings, adjusted on a sample of reporterless cells, to define cellular autofluorescence. Gating on FSC⁄SSC was used to discriminate bacteria from the background. For each sample, 20000 gated events were measured. Data were collected with the FlowMax software (Sysmex Partec, France) and analyzed with the Weasel 3.3.3 software (WEHI, USA).

To identify positive and negative populations on FL1/FL4 bi-parametric cytograms, we applied the 98% division line for each fluorescent marker, i.e. we set the threshold on the reporterless strain so that 98% of the population gave a fluorescence intensity below the threshold. Bacteria with a fluorescence signal above the threshold were considered positive. We cannot exclude that for a reporter expressed at a low level, a few positive cells might have a fluorescent intensity similar to that of the reporterless cells and be included in the negative population.

**RNA-Extraction**

2 mL aliquots were sampled from LB cultures at OD_600_=1 (exponential phase growth) and OD_600_=8 (stationary phase growth) and immediately mixed with an equal volume of RNA-later (Invitrogen, Eugene, OR, U.S.A.). Bacteria from insect cadavers were crushed in 1 mL of RNA-later and vortexed. All samples were stored at 4°C overnight to allow thorough penetration of RNA-later.

For bacteria extracted from insect cadavers, the liquid fraction was transferred to a new tube and centrifuged for 10 min at 13000 rpm at 4°C. The fat pellicle and supernatant were discarded and the pellet resuspended in 750 µL of saline. The suspension was then filtered on a cotton pad in a 1 mL syringe to remove larvae debris. Then, all samples (from *in vitro* cultures and from insect cadavers) were centrifuged for 3 minutes at 13000 rpm at 4°C and the pellets were resuspended in 1 mL of Trizol (Invitrogen, Eugene, OR, USA). Resuspended bacteria were disrupted by adding silica beads (Biospec Products, Bartlesville, OK, USA) to the suspension and shaking in a Fastprep 24 (MP Biomedicals) for 45 s at 6.5 M/s twice. The supernatant was transferred to a clean tube and 100 µL of 1-bromo-3-chloropropane (Sigma-Aldrich, Saint-Louis, MO, USA) were added. The suspension was vortexed, incubated for 10 min at room temperature and centrifuged for 15 min at 13000 rpm at 4°C. The aqueous phase was then transferred to a clean tube to which 0.1 volume of sodium acetate and 0.7 volume of isopropanol were added. The suspension was mixed with 1 µL of glycogen (Thermo Fisher Scientific, Waltham, MA, USA) and incubated at -20°C for 1 h to precipitate nucleic acids. Nucleic acids were then pelleted by centrifugation (20 min at 13000 rpm at 4°C), washed twice with 75% ethanol, air-dried and resuspended in RNase-free water (Thermo Fisher Scientific, Waltham, MA, USA). Traces of contaminating DNA were removed by TURBO⁠ DNase treatment (Invitrogen, Eugene, OR, USA).

**RNA-Seq analysis**

To assign functional categories to the differentially expressed genes, we used a database constructed by Sébastien Gélis-Jeanvoine (unpublished). This database was constructed as follows: all the genes of *B. thuringiensis* 407 were functionally re-annotated using InterProScan (1)⁠, HHSearch (2) and EggNOG (3)⁠. InterProScan v.5 was queried online through its SOAP API (http://www.ebi.ac.uk/Tools/webservices/services/pfa/iprscan5_soap) with the --goterms and --nopathways flags. GO terms were then parsed and used as three different annotations (process, function and component). HHSearch was used on our local cluster to query an HMM database prepared from the PDB70 database (2014-09-06 update), with a probability threshold of 95%. COG letters were assigned to our query genes by first aligning them with BLASTp (e-value threshold of 10^-2^) against the EggNOG v4.0 data base. The best hit for each query gene was then used to retrieve the cognate COG letter via an in-house script (EggnogGenome, <https://github.com/seb-ksl/EggnogGenome)>.

**ROS/RNS assay in 7-days post-inoculation LB cultures and LB media**

Total free radicals (reactive oxygen species or ROS and reactive nitrogen species or RNS) were quantified in 7 days post-inoculation LB media culture and LB media using the OxiSelectTM In Vitro ROS/RNS Assay Kit (Cell Biolabs, USA) according to the manufacturer’s instructions. .Bacteria grown in LB during 7 days were centrifuged at 13000 rpm for 45 sec. Supernatants and LB medium were collected, diluted 10x and incubated with the OxiSelectTM kit components in a 96 well black bottom plate during 30 min at room temperature. Fluorescence was then measured, and the results were obtained from the linear regression equation of a predetermined DCF standard curve. Measurements were performed on an Infinite M200 Pro microplate spectrofluorimeter (Tecan, USA).

**REFERENCES**

1. Jones P, Binns D, Chang H-Y, Fraser M, Li W, McAnulla C, McWilliam H, Maslen J, Mitchell A, Nuka G, Pesseat S, Quinn AF, Sangrador-Vegas A, Scheremetjew M, Yong S-Y, Lopez R, Hunter S. 2014. InterProScan 5: genome-scale protein function classification. Bioinformatics 30:1236–1240.

2. Söding J. 2005. Protein homology detection by HMM-HMM comparison. Bioinformatics 21:951–960.

3. Powell S, Forslund K, Szklarczyk D, Trachana K, Roth A, Huerta-Cepas J, Gabaldón T, Rattei T, Creevey C, Kuhn M, Jensen LJ, von Mering C, Bork P. 2014. eggNOG v4.0: nested orthology inference across 3686 organisms. Nucleic Acids Res 42:D231-239.
