# Supplementary material for: A Sporulation-Independent Way of Life for Bacillus thuringiensis in the Late Stages of an Infection
Source: mBio. 2023 Apr 27;14(3):e00371-23. doi: 10.1128/mbio.00371-23 (PMC10294645; doi:10.1128/mbio.00371-23)
Supplement: TABLE S1 [file mbio.00371-23-s0002.docx]

**Table S1.a. Plasmids used in this study.**

| **Name** | **Relevant information** | **Référence** |
| --- | --- | --- |
| pHT304 | Replicative multicopy *E. coli*/*B. thuringiensis* shuttle vector. | (1) |
| pHT304.18 | Replicative multicopy *E. coli*/*B. thuringiensis* shuttle vector. | (2) |
| pP*x’gfp_Bte_* | *B. thuringiensis* codon optimized *gfp* including 24 bp encoding the first eight amino acids of *comGA* (3), cloned in the pHT304’P*xyl+* vector with the modified RBS AGGAGG (4). | (5) |
| pHT-*gfp_Bte_AAV* | Destabilized *gfp* using *ssrA* –AAV tag designated *gfpAAV* cloned into pHT304.18. | (5) |
| pP*nprA*’*gfp_Bte_AAV* | The promoter region of the *nprA* gene was amplified by PCR from the chromosome of *B. thuringiensis* strain 407 using primer pairs P*nprA*-F-XbaI/P*nprA*-R-AscI and cloned between the XbaI and AscI restriction sites of pHT-*gfpAAV* to detect fluctuations in necrotrophism expression. | This study |
| pP*spoIIQ*_ST_ | The promoter region of the *spoIIQ* gene was amplified by PCR from the chromosome of *B. thuringiensis* strain 407 using primer pairs P*spoIIQ*-F-SphI/P*spoIIQ*-R-XbaI and cloned between the SphI and XbaI restriction sites of pHT304.18 p*PspoIIQ’*. This construction was prepared for future *mcherry* insertion and expression optimization by cloning the transcriptional terminator of phage lambda (6) using primer pairs Term-F-KpnI/Term-R-EcoRI between the KpnI and EcoRI restriction sites of p*PspoIIQ*’. We also added The STAB-SD (7) sequence by PCR amplification using primer pairs StabSD-F-XbaI/StabSD-R-BamHI followed by cloning between the XbaI and BamHI restriction sites of pP*spoIIQ*’TermLambda. This generated pP*spoIIQ*_ST_. | This study |
| pP*spoIIQ*’*mcherry* | *B. thuringiensis* codon optimized *ComGAmcherry* was amplified by PCR from Pp*x+*’*comGAmcherry* (unpublished) using primer pairs comGAmC-F-BamHI/mC-R-KpnI and cloned between the BamHI and KpnI restriction sites of pP*spoIIQ*_ST_ to create a transcriptional fusion to assess sporulation. |  |
| pP*spoIIQ*’*mcherry*-P*nprA*’*gfp_Bte_AAV* | P*spoIIQ* was amplified by PCR from pP*spoIIQ*_ST_ using primer pairs P*spoIIQ*-F-SalI/Term-R-SphI and cloned between the SalI and SphI restriction sites of pP*nprA*’gfp*_Bte_*AAV. This construction is used to monitor sporulation and transient necrotrophism expression. | This study |
| pP*x+*’*gfp_BteT_* | The transcriptional terminator of phage lambda was amplified by PCR using primer pairs Term-F-KpnI/Term-R-AscI-XbaI-EcoRI and cloned between the KpnI and EcoRI restriction sites of pP*x+*’*gfp_Bte_* to obtain pP*x+*’*gfp_BteT_* | This study |
| pP*x+*’*gfp_Bte_*-P*spoIIQ*’*mcherry* | P*spoIIQ* was amplified by PCR from pP*spoIIQ*_ST_ using primer pairs P*spoIIQ*-F-AscI/Term-R-EcoRI and cloned between the AscI and EcoRI restriction sites of pP*x+*’*gfp_BteT_*. This construct was used to detect GFP production upon xylose induction among the non-sporulating cells. | This study |
| p*PspoIIQ*’*mcherry*-P*ykuN2*’*gfp_Bte_AAV* | The promoter region of the *ykuN2* gene was amplified by PCR from the chromosome of *B. thuringiensis* strain 407 using primer pairs P*ykun2*-F-SalI/P*ykun*2-R-AscI and cloned between the SalI and AscI restriction sites of pP*spoIIQ*’*mCherry*-P*nprA*’*gfp_Bte_AAV*. This construction was used to determine the promoter activity of *ykuN2* among the non-sporulating cells. | This study |
| pP*spoIIQ*’*mcherry*-P*isdE1*’*gfp_Bte_AAV* | This plasmid was constructed as above with the promoter region of *isdE1* amplified using primer pairs P*isdE1*-F-SalI/P*isdE1*-R-AscI to determine the promoter activity of *isdE1* among the non-sporulating cells. | This study |
| pP*spoIIQ*’*mcherry*-P*dhbA*’*gfp_Bte_AAV* | This plasmid was constructed as above with the promoter region of *dhbA* amplified using primer pairs P*dhbA*-F-SalI/P*dhbA*-R-AscI to determine the promoter activity of *dhbA* among the non-sporulating cells. | This study |
| pP*spoIIQ*’*mcherry*-P*BTB_c10430*’*gfp_Bte_AAV* | This plasmid was constructed as above with the promoter region of *BTB_c10430* amplified using primer pairs P*BTB_c10430*-F-SalI/P*BTB_c10430*-R-AscI to determine the promoter activity of *BTB_c10430* among the non-sporulating cells. | This study |
| pP*spoIIQ*’*mcherry*-P*katE1*’*gfp_Bte_AAV* | This plasmid was constructed as above with the promoter region of *katE1* amplified using primer pairs P*katE1*-F-SalI/P*katE1*-R-AscI to determine the promoter activity of *katE1* among the non-sporulating cells. | This study |
| pP*spoIIQ*’*mcherry*-P*sodA1*’*gfp_Bte_AAV* | This plasmid was constructed as above with the promoter region of *sodA1* amplified using primer pairs P*sodA1*-F-SalI/Ps*odA1*-R-AscI to determine the promoter activity of *sodA1* among the non-sporulating cells. | This study |

**Table S1.b. Strains used in this study.**

| **Name** | **Relevant information** | **Référence** |
| --- | --- | --- |
| Bt (pHT304) | *B. thuringiensis* 407^-^ carrying the empty pHT304 vector and used as a fluorescence^-^ control. | (5) |
| Bt (pP*spoIIQ*’*mcherry*-P*nprA*’*gfp_Bte_AAV*) | *B. thuringiensis* strain 407^-^ in which we measure the activity of the promoter of *nprA*, using a reporter gene encoding an unstable GFP, as well as the activity of the promoter of *spoIIQ*, using mCherry. | This study |
| Bt (pP*spoIIQ*’*mcherry*) | *B. thuringiensis* strain 407^-^ in which we measure the activity of the promoter of *spoIIQ*, using a reporter gene encoding mCherry. | This study |
| Bt (pP*x+*’*gfp_Bte_*-P*spoIIQ*’ *mcherry*) | *B. thuringiensis* strain 407^-^ in which we measure the activity of the promoter of *spoIIQ,* using a reporter gene encoding mCherry associated to the transcriptional fusion between P*xyl*+ and GFP to determine GFP synthesis ability upon xylose induction. | This study |
| Bt (p*PspoIIQ*’*mcherry*-P*ykuN2*’*gfp_Bte_AAV*) | *B. thuringiensis* strain 407^-^ in which we measure the activity of the promoter of *ykun2*, using a reporter gene encoding an unstable GFP, as well as the activity of the promoter of *spoIIQ*, using mCherry. | This study |
| Bt (pP*spoIIQ*’*mcherry*-P*isdE1*’*gfp_Bte_AAV*) | Same as the strain above with the promoter region of *isdE1*. | This study |
| Bt (pP*spoIIQ*’*mcherry*-P*dhbA*’*gfp_Bte_AAV*) | Same as the strain above with the promoter region of *dhbA*. | This study |
| Bt (pP*spoIIQ*’*mcherry*-P*BTB_c10430*’*gfp_Bte_AAV*) | Same as the strain above with the promoter region of *BTB_c10430*. | This study |
| Bt (pP*spoIIQ*’*mcherry*-P*katE1*’*gfp_Bte_AAV*) | Same as the strain above with the promoter region of *katE1*. | This study |
| Bt (pP*spoIIQ*’*mcherry*-P*sodA1*’*gfp_Bte_AAV*) | Same as the strain above with the promoter region of *sodA1*. | This study |

**Table S1.c. Oligonucleotides used in this study.**

| **Name** | **Sequence** |
| --- | --- |
| PnprA-F-XbaI | gctctagaGCCGGAAAGGGTTTTTTCAATATTTG |
| PnprA-R-AscI | tggcgcgccGCTTTCTTACCAGTCGCTCC |
| PspoIIQ-F-SphI | acatgcatgcGCATCTTCGGTTGAAGTTCTAC |
| PspoIIQ-R-XbaI | gctctagaCATCACCTCAGCAATCATTTTGAAC |
| Term-F-KpnI | ggggtaccGATCTCTGCAGTCGCGATGATTAATTAATTC |
| Term-R-EcoRI | ggaattcCGCAACGTTCTTGCCATTGCTGC |
| StabSD-F-XbaI | gctctagaTCTTGAAAGGAGGGATGCCTAAAAA |
| StabSD-R-BamHI | cgggatccATAAAATGATTTTTCATAAATCCA |
| comGAmC-F-BamHI | cgggatccTTAAGGAGGTGACACCATGAATGGG |
| mC-R-KpnI | ggggtaccTTACTTATATAATTCATCCATTCCAC |
| PspoIIQ-F-SalI | acgcgtcgacGCATCTTCGGTTGAAGTTCTAC |
| Term-R-SphI | acatgcatgcGCAACGTTCTTGCCATTGCTGC |
| Term-R-AscI-XbaI-EcoRI | ggaattccgctctagaggcgcgccGCAACGTTCTTGCCATTGCTGC |
| PspoIIQ-F-AscI | tggcgcgccGCATCTTCGGTTGAAGTTCTAC |
| Pykun2-F-SalI | acgcgtcgacAAAAAAGCACAGATGATTGTATAGT |
| Pykun2-R-AscI | tggcgcgccCATCTAAACTAACTTTAATTAAATC |
| PisdE1-F-SalI | acgcgtcgacTATTGTTAACTAGAGCGCGGCGAAA |
| PisdE1-R-AscI | tggcgcgccAGACGCTTTCTCGTCCCCTTTGGCA |
| PdhbA-F-SalI | acgcgtcgacTATACAAATCTTCTATAACACTATG |
| PdhbA-R-AscI | tggcgcgccCTAAAAACATTTTGGCAACAACACT |
| PBTB_c10430-F-SalI | acgcgtcgacTAAATGCAATTTGGCAACAAACTAA |
| PBTB_c10430-R-AscI | tggcgcgccCGTAAGCATAGTACTGCTTAAATAA |
| PkatE1-F-SalI | acgcgtcgacCGAAAAGAATTATCTTAAAAGCCAA |
| PkatE1-R-AscI | tggcgcgccCTTGGTTTGTTGTTAAAGCATGTTT |
| PsodA1-F-SalI | acgcgtcgacCATATCCATTTCGCATGTTTATTA |
| PsodA1-R-AscI | tggcgcgccGGATGTTCATTGTTTCTTTGTCCAA |

Pink letters indicate enzymatic restriction sites.

**REFERENCES**

1. Arantes O, Lereclus D. 1991. Construction of cloning vectors for *Bacillus thuringiensis*. Gene 108:115–119.

2. Agaisse H, Lereclus D. 1994. Structural and functional analysis of the promoter region involved in full expression of the *cryIIIA* toxin gene of *Bacillus thuringiensis*. Mol Microbiol 13:97–107.

3. Veening J-W, Smits WK, Hamoen LW, Jongbloed JDH, Kuipers OP. 2004. Visualization of differential gene expression by improved cyan fluorescent protein and yellow fluorescent protein production in *Bacillus subtilis*. Appl Environ Microbiol 70:6809–6815.

4. Stammen S, Müller BK, Korneli C, Biedendieck R, Gamer M, Franco-Lara E, Jahn D. 2010. High-yield intra- and extracellular protein production using *Bacillus megaterium*. Appl Environ Microbiol 76:4037–4046.

5. Ben Rejeb S, Lereclus D, Slamti L. 2017. Analysis of abrB Expression during the Infectious Cycle of *Bacillus thuringiensis* Reveals Population Heterogeneity. Front Microbiol 8:2471.

6. Scholtissek S, Grosse F. 1987. A cloning cartridge of lambda t(o) terminator. Nucleic Acids Res 15:3185.

7. Agaisse H, Lereclus D. 1996. STAB-SD: a Shine-Dalgarno sequence in the 5’ untranslated region is a determinant of mRNA stability. Mol Microbiol 20:633–643.
